# Cognitive Testing in Spanish Older Adults: A Scoping Review

**DOI:** 10.3390/geriatrics11020045

**Published:** 2026-04-10

**Authors:** Lucía Sáez-González, Luis A. Martínez, Gema Blázquez-Abellán, José Antonio Carbajal de Lara, Rosa M. Martinez-Garcia, Lucía Castro-Vázquez

**Affiliations:** 1 NUTRISAF Research Group, Faculty of Pharmacy, University of Castilla-La Mancha (UCLM), Avda. Doctor Jose María Sanchez Ibañez s/n, 02008 Albacete, Spain; luisa.martinez@uclm.es (L.A.M.); gemma.blazquez@uclm.es (G.B.-A.); josea.carbajal@uclm.es (J.A.C.d.L.); luciaisabel.castro@uclm.es (L.C.-V.); 2 NUTRISAF Research Group, Faculty of Nursing, University of Castilla-La Mancha (UCLM), Camino Pozuelo s/n, 16071 Cuenca, Spain; rosamaria.martinez@uclm.es

**Keywords:** neuropsychological test, mild cognitive impairment, screening, dementia, healthy aging, mental health

## Abstract

**Abstract:**

**Background/Objectives:** Cognitive impairment is a major concern in aging populations. Early detection through validated neuropsychological tests is essential for dementia risk stratification and preventive interventions. This scoping review (PRISMA-ScR, 2013–2023, registration protocol: 10.17605/OSF.IO/8NHJF) evaluated cognitive testing trends in aging research and identified the most frequently used neuropsychological screening tests in Spanish populations. **Methods:** Searches in PubMed and Web of Science (March 2024) yielded 730 records; 156 were reviewed in full, and 15 met inclusion criteria for Spanish adults ≥65 years. **Results:** The Mini-Mental State Examination was the most-used test, followed by verbal fluency and Trail Making Test. No test covered all six DSM-5 cognitive domains, and social cognition was never assessed in any of the studies. The Montreal Cognitive Assessment was underused despite its superior sensitivity. **Conclusions:** Findings support developing a tailored, multidomain battery combining global and domain-specific tests. Social cognition assessments should be included to ensure a complete cognitive domain coverage.

**Key Findings:**

- The Mini-Mental State Examination (MMSE) was used in 100% of studies examining Spanish older adults ≥65 years reflecting its dominant role in Spanish cognitive aging research despite its documented low sensitivity for mild cognitive impairment (MCI).
- Multidomain cognitive coverage was limited. No identified neuropsychological test assessed all six DSM-5 cognitive domains.
- Social cognition was never assessed in any of the included studies, representing a significant gap in comprehensive cognitive evaluation.
- The Montreal Cognitive Assessment (MoCA) was underrepresented despite international evidence supporting its sensitivity for MCI detection.
- Our findings underscore the need to design multidomain, context-adapted screening batteries for Spanish older adults that incorporate social cognition.

## 1. Introduction

Driven by rising life expectancy, population aging poses global social, economic and environmental challenges [1]. Mild cognitive impairment (MCI) and dementia substantially impact functional independence, quality of life, and healthcare costs.

Cognitive function in older adults is conceptualized as existing along a continuum. One end of this spectrum corresponds to normal cognitive aging, which typically involves slight reductions in processing speed and specific memory domains that are viewed as components of healthy aging. The other end represents dementia, marked by cognitive deficits significant enough to meaningfully impair autonomy in everyday activities. MCI occupies an intermediate position, representing a level of impairment greater than expected for age and educational background but not severe enough to meet the criteria for dementia [2].

A key aspect in the conceptualization of cognitive impairment is the distinction between its subjective and objective forms. “Subjective” refers to self-reported deterioration in memory or thinking that precedes detectable deficits on standardized cognitive tests; although individuals report subjective memory complaints (SMC), their neuropsychological performance remains within normal limits. In contrast, objective MCI is identified through measurable deficits on standardized neuropsychological assessments. These intersecting concepts collectively conceptualize cognitive impairment as a heterogeneous, bidirectional process in which individuals may progress to dementia, remain stable, or even revert to normal cognition, underscoring the importance of early detection and screening [3,4]. The prognostic significance of SMC varies considerably; approximately 25–50% of individuals with SMC show progression to MCI within 10 years, with faster progression observed in those with concurrent Alzheimer’s disease biomarkers.

**Figure 1:**
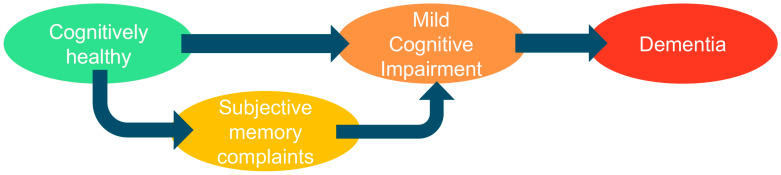
Stages of the cognitive continuum: cognitively healthy individuals, subjective memory complaints (not universally present), mild cognitive impairment, and dementia.

These successive stages of cognitive continuum are illustrated in Figure 1.

Neuropsychological tests are fundamental instruments for detecting MCI in both clinical and research contexts. Within research settings, these tests serve multiple functions: identifying individuals with MCI, characterizing risk profiles for dementia development, and evaluating the effectiveness of preventive interventions against Alzheimer’s disease and other dementias [5]. The diversity of instruments—each with distinct psychometric properties, validation samples and cognitive focus—poses challenges for selection aligned with research objectives [6].

It is crucial to distinguish between cognitive screening and comprehensive neuropsychological assessment. Cognitive screening aims to rapidly detect possible cognitive difficulties that may require further neuropsychological assessment to confirm a diagnosis. Cognitive screening is performed through brief neuropsychological tests, designed to be administered by a range of health professionals in primary care or community settings. In contrast, a full neuropsychological assessment is an in-depth evaluation carried out by clinical specialists, usually lasting several hours and integrating multiple neuropsychological tests, behavioral observations and contextual information. The present scoping review focuses on the use of neuropsychological tests within studies conducted in Spain, either for screening or extensive neuropsychological assessment purposes.

**Figure 2:**
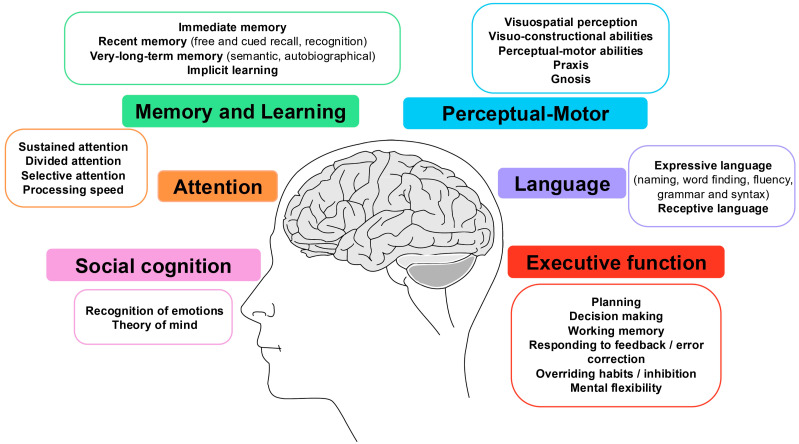
The six major cognitive domains and subdomains as defined by DSM-5: social cognition, complex attention, memory and learning, perceptual–motor function, language, and executive function.

To organize the heterogeneous array of cognitive screening instruments, we adopted the Diagnostic and Statistical Manual of Mental Disorders, Fifth Edition (DSM-5) neurocognitive framework as a conceptual reference. This framework is widely used in clinical neuropsychology and geriatric psychiatry and offers a comprehensive, internationally recognized classification of cognitive domains relevant to mild and major neurocognitive disorders. The DSM-5 defines six major cognitive domains—social cognition, complex attention, memory and learning, perceptual–motor function, language, and executive function—each encompassing specific subdomains [7]. These domains represent international standards in neuropsychological classification and provide a comprehensive framework for systematic cognitive assessment. Figure 2 presents the six primary cognitive domains and their constituent subdomains according to DSM-5 criteria.

Different types of dementia characteristically affect distinct cognitive domains, and the pattern of cognitive impairment evolves across disease stages. In the early stages of MCI due to probable Alzheimer’s disease, decline predominantly affects memory and learning; as the disease progresses, additional cognitive domains become compromised; in severe Alzheimer’s disease, widespread cognitive deterioration occurs across multiple domains, resulting in dependence in activities of daily living and increased frailty. This heterogeneity in cognitive profiles underscores the need for comprehensive, multidomain neuropsychological evaluation.

To ensure complete cognitive evaluation aligned with research aims, comprehensive screening of all potentially affected cognitive domains is essential. A scoping review of the cognitive screening and neuropsychological assessment literature can provide a comprehensive overview of current research practices, identify the most frequently utilized instruments in specific populations, and inform evidence-based selection of tests that collectively capture the full spectrum of cognitive function while remaining feasible for administration. This is particularly relevant for the Spanish population, where standardized guidance on the neuropsychological test selection for cognitive screening remains limited. Although several cognitive screening instruments have been translated into Spanish, published evidence indicates significant gaps in culturally validated normative data, heterogeneous cutoff scores across Latin American countries and Spain, and limited guidance regarding optimal test batteries for specific Spanish-speaking populations aged 65 years and older [8,9].

In the context of population aging, identifying modifiable risk factors for dementia and promoting protective factors is essential to support healthy aging and preserve quality of life in older adults [10]. Therefore, this scoping review examined the most frequently utilized neuropsychological screening instruments in Spanish populations aged 65 years and older to provide evidence-based guidance for selecting appropriate cognitive assessment tools.

Several systematic reviews and meta-analyses have evaluated the diagnostic accuracy and normative data of individual tools for detecting MCI and dementia, often across heterogeneous international samples [11,12,13,14]. However, less attention has been paid to real-world implementation patterns within specific national contexts. These quantitative syntheses do not specifically map which cognitive screening instruments are being implemented in research involving Spanish older adults, nor how these instruments collectively cover the DSM-5 cognitive domains. In contrast to prior systematic reviews and meta-analyses, our scoping review aims to map how these instruments are implemented in aging research in Spain—across population segments and cognitive domains—by systematically charting instrument use and DSM-5 domains coverage, thereby identifying implementation gaps between available evidence and routine practice, and providing pragmatic guidance for selecting context-appropriate screening batteries.

## 2. Objectives

This scoping review aimed to identify the most commonly used neuropsychological tests for screening cognitive impairment in Spanish community-dwelling older adults aged 65 years and older from 2013 to 2023. The research question was formulated following the PICO(S) strategy: (i) Participants: non-institutionalized Spanish adult population; (ii) Intervention characteristics: use of neuropsychological tests; (iii) Outcome: evaluation of CI; (iv) Study design: observational cross-sectional and longitudinal studies and randomized controlled trials (RCT) published between 2013 and 2023.

The primary objective was to identify patterns of instrument use in real-world research settings. Secondary objectives included characterizing (i) cognitive domains assessed by each instrument, classified according to the six major DSM-5 neurocognitive domains; (ii) describing administration characteristics and feasibility considerations; and (iii) identifying potential gaps in multidomain cognitive coverage. The review was not designed to evaluate diagnostic accuracy or comparative psychometric superiority of the included instruments. The synthesis of this information would support an evidence-based test selection for cognitive screening batteries tailored to the Spanish population.

## 3. Materials and Methods

This scoping review was conducted in accordance with the PRISMA-ScR guidelines (Page et al., 2021) [15]. The protocol was developed prior to study initiation and is registered in the Open Science Framework (Registration protocol: 10.17605/OSF.IO/8NHJF). The completed PRISMA-ScR checklist is provided in the Supplementary Material. A literature search was performed through two different databases, Web of Science (WOS) and PubMed, and studies meeting eligibility criteria were selected.

### 3.1. Eligibility Criteria

Inclusion criteria were developed a priori using the PICO(S) framework to ensure systematic and reproducible study selection. The following inclusion criteria were applied: (i) Participants: non-institutionalized Spanish adults aged ≥65 years; (ii) Intervention: use of neuropsychological tests, operationalized as standardized cognitive assessments administered directly to participants that measure performance in one or more specific cognitive domains through objective performance-based measures; (iii) Outcome: evaluation or measurement of cognitive impairment; (iv) Study Design: observational studies (cross-sectional and longitudinal) and RCT. Studies published in English or Spanish between 1 January 2013 and 31 December 2023 were included.

The restriction of the population to older adults living in Spain provides context-specific evidence that can directly inform cognitive screening practices in Spanish primary care and aging research. Including studies from other Spanish-speaking countries would have introduced marked heterogeneity in healthcare systems, educational backgrounds, and available normative data, reducing the applicability of the findings to the Spanish setting. The 2013–2023 time window was chosen to capture contemporary cognitive screening practices aligned with current diagnostic criteria for MCI and dementia and with the DSM-5 framework for cognitive domains. Limiting inclusion to English or Spanish-language publications ensured accurate interpretation of study methods and results and reflects the main languages of scientific output in Spain. While this restriction may have led to the omission of a small number of studies, it is unlikely to have materially affected identification of the core cognitive screening instruments used in Spanish older adults.

Studies were excluded if they met any of the following criteria: (i) did not evaluate Spanish older adults living in Spain; (ii) did not employ neuropsychological tests; (iii) included participants with primary diagnoses of chronic pain, fibromyalgia, stroke, or neurodegenerative conditions other than Alzheimer’s disease or other dementias (e.g., multiple sclerosis, Parkinson’s disease); (iv) included only informant or caregiver respondents rather than direct participant assessment; (v) were reviews, opinion papers, editorials, or commentaries; or (vi) were solely validation or normative data studies without original cognitive assessment data.

The rationale for excluding certain neurodegenerative conditions (multiple sclerosis, Parkinson’s disease, stroke) was to focus on studies examining cognitive impairment related to primary aging processes or pathologies, rather than those with cognitive impairment secondary to specific neurological diseases with distinct pathophysiological mechanisms.

### 3.2. Search Strategy and Study Selection

A comprehensive literature search was conducted independently by both primary researchers (Sáez-González, L. and Martínez-López, L.A.) in March 2024, using identical search strategies across two databases: PubMed and Web of Science. The search retrieved articles indexed from 1 January 2013 to 31 December 2023 (10-year period). The search strategy combined multiple relevant terms and their truncations using Boolean operators: neuropsychol*, cognit*, neurocognit*, mental, brain, assessment, test*, “screening tool”, questionnaire, Alzheimer, “cognitive impairment”, “cognitive decline”, “cognitive function”, dementia, and Spanish. The complete search strategies are provided in the Supplementary Material.

The study selection process followed a two-stage approach with independent dual review:

1. Title and Abstract Screening: Researchers independently screened all titles and abstracts of the 730 unique articles against the eligibility criteria using standardized screening forms. Reference lists of included articles and previously published systematic reviews and meta-analyses on cognitive screening were manually reviewed to identify potentially relevant studies missed by the database search. This stage resulted in 156 articles identified as potentially eligible for full-text review.
2. Full-Text Review: The complete texts of the 156 articles were independently assessed for eligibility. Access to full-text articles was obtained for 139 studies (88.6%), which were then read and evaluated against inclusion/exclusion criteria. Fourteen articles (8.9%) could not be accessed despite contact attempts with the corresponding authors. Disagreements between the two independent reviewers were resolved through discussion and consensus with a third reviewer (Castro-Vázquez, L.). Following full-text review, 47 articles met all inclusion criteria and were included in the scoping review. Of these 47 studies, 15 articles specifically examined populations aged ≥65 years of age and represent the primary focus of the review, aligning with the study objective of providing guidance for cognitive assessment in older adults. The remaining 32 studies examined mixed-age populations including younger participants. All 47 studies were retained to maximize comprehensiveness of instrument characterization, allowing stratified analyses between populations aged ≥65 years and mixed-age groups (see Supplementary Material).

### 3.3. Data Extraction

Data extraction was performed independently by both primary researchers using a standardized form. The standardized extraction form captured (i) study characteristics (author, publication year, county/region); (ii) study design (cross-sectional vs. longitudinal; observational vs. RCT); (iii) sample characteristics (sample size, age range/mean, sex distribution, educational level, cognitive status of participants); (iv) neuropsychological instrument(s); (v) cognitive domains assessed classified according to DSM-5 framework; (vi) administration time; and (vii) study outcomes and findings regarding instrument performance.

The principal characteristics of each identified neuropsychological test—including frequency of use across included studies, cognitive domains assessed, administration time, psychometric properties, and limitations—were synthesized into tabular format for comparison and analysis. This charting approach facilitated systematic comparison of studies and instruments, identified patterns in test utilization, enabled characterization of cognitive domain coverage across instruments, and provided the foundation for synthesizing recommendations regarding test selection. Cognitive domains were classified according to the six major DSM-5 domains [7]. The DSM-5 framework was selected because it provides an internationally recognized and clinically operational taxonomy of cognitive functioning, facilitating standardized categorization across heterogeneous instruments. Domain assignment for each test was based on the primary cognitive functions targeted by its core tasks, as described in original validation studies. We acknowledge that most instruments were not originally validated to align specifically with DSM-5 domains, and that many tests tap multiple domains simultaneously. Accordingly, domain classification in this review should be interpreted as a pragmatic mapping intended to highlight areas of under-representation, rather than as a strict one-to-one correspondence between individual tests and DSM-5 domains.

In line with PRISMA-ScR guidelines for scoping reviews, formal risk-of-bias or diagnostic-accuracy assessments were omitted. This exploratory focus prioritizes mapping patterns over quality appraisal. Readers should interpret findings as descriptive, not comparative; see limitations in Section 6, Limitations.

## 4. Results and Discussion

This scoping review provides a complementary perspective to prior systematic reviews and meta-analyses that focused on diagnostic accuracy of individual instruments in MCI and dementia by mapping which cognitive tests are actually used in research studies conducted in Spain and how they cover the DSM-5 cognitive domains. Because we did not perform a formal risk-of-bias assessment or extract diagnostic-accuracy indices, our findings should not be interpreted as comparative evidence of test performance. Rather, they describe implementation patterns and DSM-5 domain coverage in Spanish older adults, which should be integrated with previous diagnostic-accuracy reviews.

**Figure 3:**
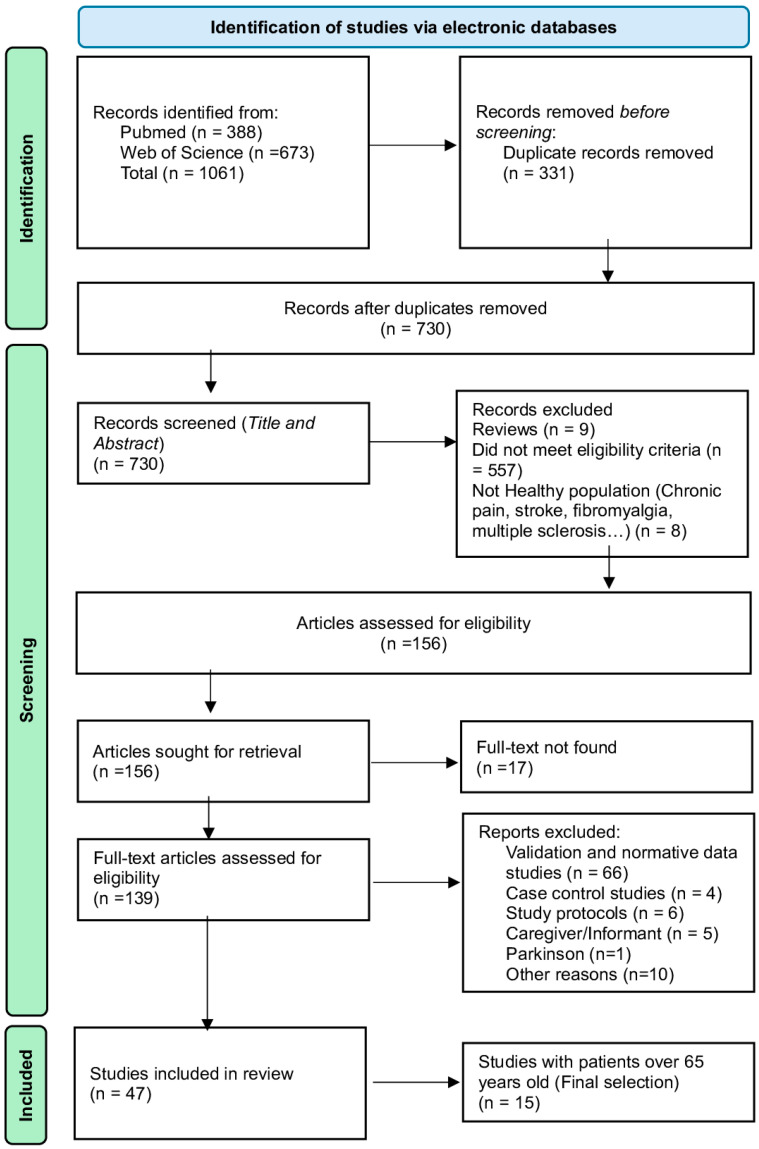
PRISMA flow diagram showing the studies included and excluded (Page et al., 2021) [15].

Figure 3 illustrates the selection process. The initial search yielded 1061 results: 388 from PubMed and 673 from Web of Science. After automated duplicate removal, the search strategy yielded 730 unique articles, of which 156 underwent full-text screening. Following detailed review, 47 studies met all eligibility criteria for examining cognitive assessment in Spanish samples. Of these 47 studies, 15 specifically examined populations aged 65 years or older and represent the primary focus of this review. The remaining 32 studies provided a broader context regarding instruments used in mixed-age Spanish populations and may be relevant for older adults if age-adjusted norms exist.

Table 1 presents a comprehensive summary of the 15 studies examining older populations (≥65 years), including author and publication year, placement, objectives, study design, detailed characteristics of the sample (size, age, sex distribution), neuropsychological instruments employed, covariates, and main outcomes and findings. See the Supplementary Material for a complete overview of the review results.

### 4.1. Study Characteristics and Design Distribution

**Figure 4:**
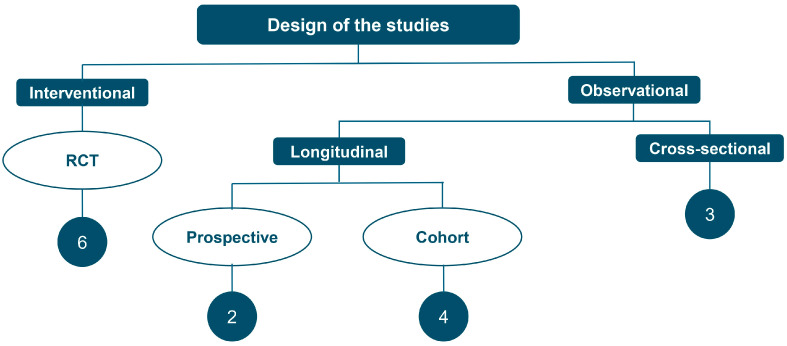
Distribution of the 15 included studies by study design: randomized controlled trials (*n* = 6, 40%), cross-sectional studies (*n* = 3, 20%), cohort studies (*n* = 4, 27%), and longitudinal follow-up studies (*n* = 2, 13%).

The 15 studies examining older populations comprised two major categories based on study design. Six studies were RCTs evaluating cognitive training programs, physical exercise, or lifestyle interventions on cognitive outcomes [16,20,21,24,25,26]. The remaining nine studies employed observational designs: three cross-sectional studies [16,17,27] four prospective cohort studies [17,22,23,28], and two longitudinal follow-up studies [27,30]. Figure 4 displays the distribution of studies by design type.

The predominance of observational designs (60%) reflects the field’s focus on epidemiological questions regarding cognitive impairment prevalence, risk factors, and natural history in community-dwelling Spanish older adults. While observational designs effectively generate evidence about real-world cognitive screening practices and naturally occurring cognitive change, they provide limited evidence regarding comparative effectiveness or superiority of different neuropsychological instruments. Moreover, these observational studies primarily relied on global screening instruments, particularly the Mini-Mental State Examination (MMSE), rather than comprehensive neuropsychological batteries, potentially limiting detection of domain-specific cognitive vulnerabilities and their associated risk factors.

All six RCTs employed the MMSE as their primary cognitive outcome, likely reflecting its established use and familiarity among Spanish researchers. While this consistency facilitates within-Spanish-literature comparisons, it limits cross-cultural synthesis with international trials that increasingly employ more sensitive instruments such as the Montreal Cognitive Assessment (MoCA) or computerized cognitive batteries [31].

### 4.2. Patterns Across the Cognitive Continuum

**Figure 5:**
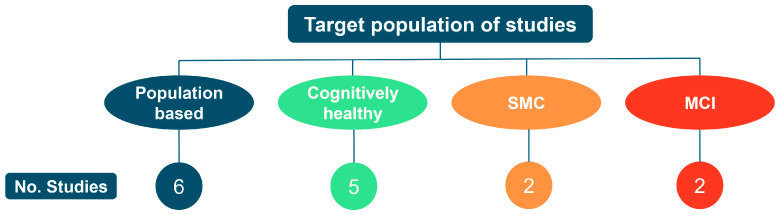
Distribution of the 15 included studies by target population: population-based (*n* = 6, 40%), cognitively healthy older adults (*n* = 5, 33%), individuals with subjective memory complaints (*n* = 2, 13%), and patients with mild cognitive impairment (*n* = 2, 13%).

The included studies encompassed diverse populations across the full spectrum of cognitive aging. Studies examined community-based populations (*n =* 6 studies); cognitively healthy older adults recruited for specific characteristics such as physical activity level or frailty status (*n =* 5); individuals with SMC (*n* = 2,); and patients diagnosed with MCI (*n* = 2). Figure 5 illustrates this distribution.

Approaches varied systematically according to population cognitive status. In community-based samples, neuropsychological tests functioned primarily as screening instruments to detect global MCI or cognitive changes associated with frailty, multimorbidity, lifestyle behaviors (e.g., physical activity, Mediterranean diet adherence), or environmental exposures (e.g., second-hand smoke) [16,17,20,22,23,30]. These studies typically employed single global screening instruments—MMSE—reflecting practical constraints of large epidemiological studies where brief, feasible assessments are essential.

In contrast, studies examining individuals with SMC or diagnosed MCI employed substantially more comprehensive assessment strategies. These investigations combined global screening instruments with multiple domain-specific tests targeting memory, attention, and executive functions to characterize detailed cognitive profiles and monitor longitudinal changes with greater precision [18,19,21,24,26]. Studies focusing on SMC populations combined global screening (MMSE) with verbal fluency measures—both semantic and phonemic variants—to examine nuanced performance differences by sex and investigate relationships between subjective complaints and objective cognitive performance, mood status, and lifestyle variables [18,19,26]. This approach recognizes that SMC may represent a preclinical stage of cognitive impairment in some individuals, while reflecting depression, anxiety, or personality factors in others, necessitating further multidimensional assessment beyond global cognition scores.

MCI samples received the most extensive evaluations, using a greater number of tests, with studies typically employing comprehensive batteries to capture subtle changes in episodic memory, attention, and executive functioning, cognitive domains particularly relevant for predicting progression from MCI to dementia [21,24,25]. The most comprehensive battery identified was employed by Bisbe et al. (2020) [21] in their RCT comparing choreographed exercise with conventional physical therapy in amnestic MCI (*n* = 31 participants). This battery included MMSE, Trail Making Test (TMT) parts A and B, semantic and phonemic verbal fluency (SVF and PVF), Boston Naming Test (BNT), Judgment of Line Orientation Test (JLOT), and multiple Wechsler Memory Scale-III (WMS-III) subtests, providing coverage across five of the six DSM-5 cognitive domains.

These differential patterns of use of neuropsychological tests across different studies populations reflect appropriate tailoring of evaluation depth to population characteristics and study objectives. However, this variability also complicates cross-study comparisons and methodological standardization in cognitive aging research.

### 4.3. Neuropsychological Test Utilization, Frequency and Characteristics

To provide guidance for neuropsychological test selection, Table 2 details the characteristics of the six most frequently used cognitive assessment tests identified across our review. This table is specifically designed as a practical reference tool for researchers and clinicians selecting appropriate cognitive assessments. Table 2 includes for each test (1) the frequency of use in the review literature (number of articles employing this test); (2) a brief test description; (3) the administration method and time required for administration; (4) the cognitive domains assessed by each test; (5) the target population and (6) the specific limitations relevant to test interpretation. The MMSE emerged as the dominant neuropsychological instrument, appearing in all 15 studies in the age-restricted sample and in 43 of 47 studies in the full sample (see Supplementary Material). This universal adoption reflects the MMSE’s historical position as the standard cognitive screening instrument in Spanish research and clinical practice for over forty years since its initial development. Verbal fluency (VF) tests—including both semantic (e.g., animal naming) and phonemic (e.g., words beginning with specific letters) variants—ranked second in frequency, appearing in 18 studies. The TMT was employed in 12 studies. Additional frequently used instruments included Digit Span (DS) (11 studies), Clock Drawing Test (CDT) (6 studies), and BNT (6 studies).

It is important to emphasize that frequency of use should not be interpreted as evidence of superior psychometric quality or diagnostic accuracy. Instrument selection in research context may reflect historical adoption, familiarity among clinicians, feasibility constraints, or institutional tradition rather than empirical superiority. Therefore, the predominance of certain instruments such as the MMSE, should be understood as an implementation pattern rather than a validation-based endorsement.

### 4.4. Cognitive Domain Coverage: Gaps and Implications for Comprehensive Assessment

**Figure 6:**
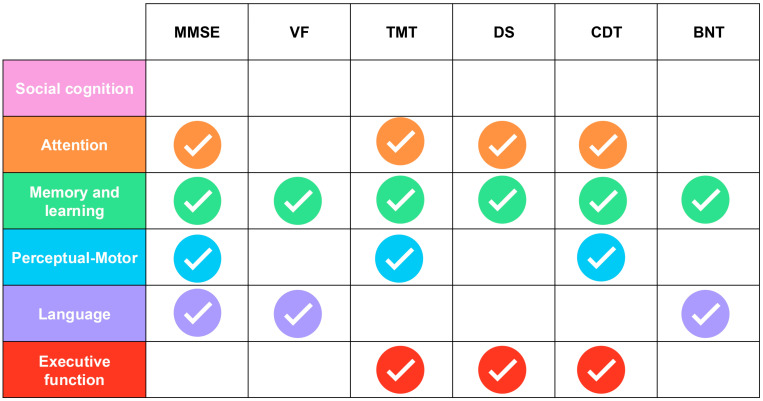
Matrix displaying the six most frequently used neuropsychological tests and their assessment coverage across the six DSM-5 cognitive domains. MMSE: Mini-Mental State Examination; VF: verbal fluency tests: TMT: Trail Making Test; DS: Digit Span; CDT: Clock Drawing Test; BNT: Boston Naming Test.

Analysis of cognitive domain coverage revealed substantial heterogeneity across individual instruments, with important implications for comprehensive cognitive assessment (Table 2). The MMSE assesses attention, language, memory and learning and perceptual-motor skills. VF tests assess language and memory. TMT assesses perceptual-motor, attention, executive function and memory and learning. DS assesses executive function, memory and learning, and attention. CDT assesses attention, memory and learning, perceptual-motor and executive function. BNT assesses language and memory and learning. Figure 6 displays the relationship between the most frequently used tests and their coverage of the six DSM-5 cognitive domains.

The widespread use of the MMSE in Spanish cognitive research reflects a set of practical strengths that have supported its continued adoption [9]. However, this prominence can mask important shortcomings that may limit its value for comprehensive cognitive assessment and for sensitively detecting early signs of impairment. The MMSE offers limited domain coverage, focusing mainly on orientation, basic memory, attention, language, and simple visuospatial skills, while providing minimal assessment of executive functions, critical for early decline and daily functioning [32], and largely omitting social cognition (Table 2). Performance is strongly influenced by noncognitive factors such as age, education, and emotional status, increasing risks of both false positives and negatives [6].

These limitations have increased interest in alternative global screeners, especially the MoCA. Although MoCA appeared in only 3 of the 47 reviewed studies, indicating limited uptake in Spanish research from 2013 to 2023, international evidence shows clear advantages for MCI detection. Multiple validation studies report higher sensitivity and specificity for MoCA than MMSE [33,34,35,36], with validation studies in Spanish populations confirming these benefits [35]. While the MoCA requires longer administration time than MMSE (approximately 15 min versus 10 min), this modest investment yields substantially broader cognitive domain assessment.

**Figure 7:**
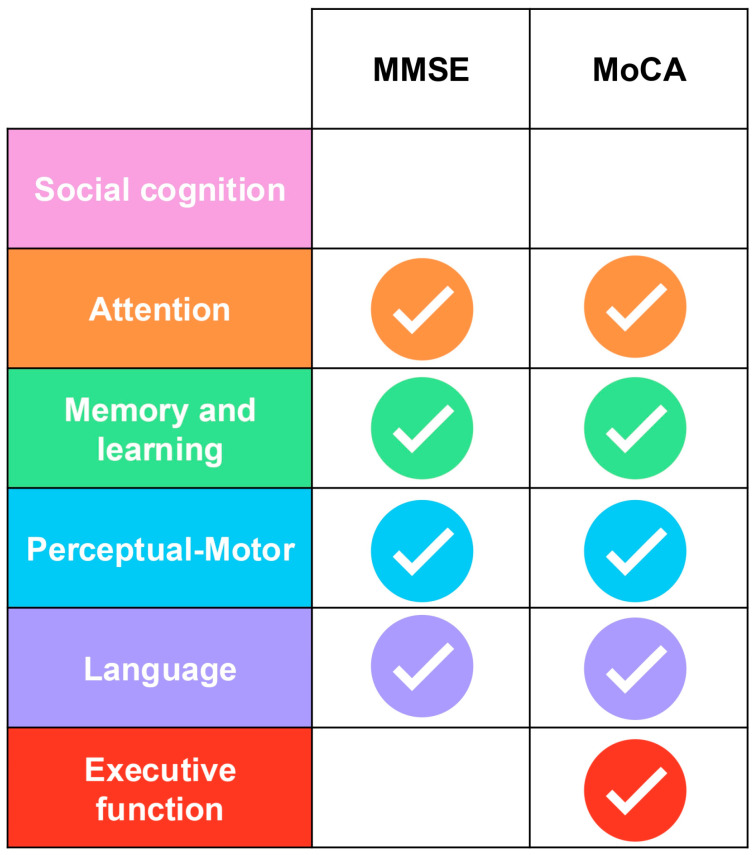
Comparative analysis of cognitive domains assessed by the Mini-Mental State Examination (MMSE) and Montreal Cognitive Assessment (MoCA) according to DSM-5 framework.

MoCA incorporates brief versions of multiple specialized neuropsychological tests, including a simplified TMT (part B) for executive function/cognitive flexibility, DS forward and backward for attention and working memory, phonemic verbal fluency for language/executive function, CDT for visuospatial function and executive planning, and delayed recall with semantic cueing for episodic memory. Figure 7 presents a comparative analysis of cognitive domains assessed by MMSE versus MoCA, illustrating MoCA’s superior domain coverage.

No identified test specifically assessed social cognition, the domain encompassing recognition of emotions, theory of mind, empathy, and interpersonal behavior regulation. This gap likely reflects the historical focus of dementia research on memory impairment and attention deficits like disorientation as primary diagnostic features, particularly in Alzheimer’s disease where episodic memory deficits represent the most prominent and earliest cognitive symptoms [37]. Social cognition impairment may be overshadowed by these more salient deficits in clinical presentation, leading to its relative neglect in standard assessment batteries.

Emerging evidence suggests that social cognition deficits appear during the MCI stage of Alzheimer’s disease and other dementias, potentially contributing to early functional difficulties, relationship strain, and reduced quality of life before memory impairment becomes severe [38,39]. Furthermore, recent epidemiological research has highlighted loneliness and social isolation—constructs intimately connected with social–cognitive abilities—as significant modifiable risk factors for dementia [40]. These findings suggest that comprehensive cognitive assessment batteries should incorporate social cognition evaluation, despite its absence from the reviewed studies.

Several instruments validated in the Spanish population could address this gap, including the Ekman 60 Faces Test for emotion recognition [41], theory of mind assessments such as false belief tasks or story-based perspective-taking measures [42], or the Inventory of Interpersonal Situations for social problem-solving [37]. Although originally developed for psychiatric populations, these instruments have been translated, adapted, and validated for Spanish-speaking individuals with Alzheimer’s disease and related dementias, supporting their feasibility for cognitive aging research.

The most comprehensive neuropsychological battery identified in the review—employed by Bisbe et al. (2020) [21] in their MCI intervention trial—illustrates one successful approach to balanced, multidomain assessment. This battery combined MMSE, TMT parts A and B, SVF, PVF, BNT, JLOT, and WMS-III subtests. This combination provided coverage across five DSM-5 domains (lacking only social cognition) while remaining feasible for administration in intervention trial contexts with total testing time of approximately 45–60 min.

This analysis suggests that cognitive screening necessarily requires strategic combination of several instruments to achieve adequate coverage across all DSM-5 domains. No single instrument—including MoCA, despite its broader coverage than MMSE—evaluates all cognitive domains. The challenge for researchers involves selecting test combinations that maximize domain coverage while minimizing total administration time and participant burden, considering factors such as population characteristics, study objectives, and resource constraints.

### 4.5. Comparison with Previous Studies

Previous studies have evaluated the diagnostic accuracy of cognitive screening tools for MCI and dementia detection. Creavin et al. (2016) [43] found the MMSE to have moderate sensitivity (71%) and specificity (89%) for dementia detection in primary care, but limited utility for MCI, highlighting its limitations for early detection. Ismail et al. (2025) [44] confirmed MoCA as an effective tool for detecting dementia, with 83% sensitivity and 82% specificity at a cutoff of 21 in US National Alzheimer Center data set. Trzepacz et al. (2015) [45] confirmed MoCA’s superior diagnostic performance compared to MMSE for MCI and Alzheimer’s disease. Pinto et al. (2019) [34] reported in their systematic review the screening superiority of MoCA compared to MMSE in the identification of MCI. The meta-analysis conducted by Wang et al. (2022) [46] also confirmed its superiority in screening dementia associated with Alzheimer’s disease from patients with MCI or healthy controls. In Spanish-speaking contexts, Custodio et al. (2020) [11] reviewed brief cognitive screenings in Latin America, reporting the popularity of the MoCA, being the most widely used test in Latin America, but also highlighting the need for cultural adaptation and different normative data.

Our scoping review complements these quantitative syntheses by examining real-world implementation trends rather than pooled diagnostic-accuracy metrics. While prior reviews establish what works best diagnostically, we map what is actually used in Spanish aging research ≥65 years: the MMSE dominates (100% of studies). Despite robust evidence of MoCA’s superior diagnostic accuracy over MMSE, and MMSE’s known limitations for MCI detection, MoCA remains underused in Spanish research, appearing in only 3 of 47 studies.

Other diagnostic-accuracy systematic reviews from various settings also highlight the need for further research on the accuracy of test combinations in screening protocols, rather than relying on an isolated one. For instance, Arevalo-Rodriguez et al. (2015) [47] could not find evidence supporting MMSE as a stand-alone single-administration test in the identification of MCI patients that could develop dementia. Although they reviewed studies with hospitalized patients, Hwang et al. (2019) [12] also could not recommend a single best instrument for cognitive screening in that context. This aligns with our recommendations to develop a screening battery of a combination of tests that balances domain coverage, administration time, and adequate sensitivity and specificity according to the target population.

No previous review has analyzed DSM-5 domain coverage across instruments in Spanish older adults. Our mapping reveals systematic gaps, including the complete absence of social cognition measures, despite growing evidence of social cognition deficits as early MCI markers. Existing reviews in Spanish-speaking populations focus on diagnostic accuracy or normative data but do not map domain coverage patterns or implementation gaps like the complete absence of social cognition assessment identified here [48,49]. Studies as the one conducted by Lee et al. (2022) [50] conclude that brief objective assessment of social cognition may enhance cognitive assessment of older adults, as the proportion of individuals classified as MCI increased by a 5% when social cognition was included in the evaluation of MCI. Furthermore, a systematic review and meta-analysis on social cognition in mild cognitive impairment and dementia conducted by Shi et al. (2025) [39] highlighted the need of including social cognition into dementia assessments as it may hold diagnostic value.

This distinction clarifies our incremental contribution: we provide pragmatic, context-specific guidance for test selection in Spanish primary care screening strategies and research settings, bridging the gap between international evidence on diagnostic performance and local implementation practices. Frequency of use does not imply diagnostic superiority—MMSE persists due to brevity (5–10 min), familiarity, and feasibility in large epidemiological studies—but highlights opportunities to adopt more sensitive tools like MoCA alongside domain-specific screeners (e.g., verbal fluency, TMT) to achieve feasible multidomain coverage, including social cognition.

## 5. Conclusions

This review identified the main neuropsychological tests used to screen cognitive impairment in the research setting. The findings highlight a predominance of brief global screening instruments and limited multidomain coverage, particularly regarding social cognition. These patterns may inform future research aimed at optimizing cognitive assessment strategies; however, recommendations should be interpreted cautiously, as this review did not evaluate comparative diagnostic accuracy or methodological quality of included studies. Screening batteries specifically designed and validated for Spanish aging populations remain underdeveloped in terms of cognitive domain coverage. Future research should prioritize development of standardized and culturally appropriate cognitive assessment protocols tailored to the Spanish population, with particular attention to low-education groups who may be disadvantaged by instruments normed primarily on highly educated samples. Such batteries should incorporate co-norming across tests to enable profile analysis, provide age- and education-stratified normative data spanning the full adult age range, and include longitudinal validation demonstrating sensitivity to cognitive change.

To ensure adequate cognitive screening, researchers should employ a set of complementary tests that collectively assess distinct cognitive domains. This recommendation specifically highlights that no single instrument adequately assesses all six DSM-5 cognitive domains; the optimal test combination depends on specific research objectives, population characteristics, and available time. MoCA + social cognition measure may represent a potentially evidence-based approach for comprehensive yet feasible assessment.

The ideal battery should (i) assess all six DSM-5 cognitive domains to enable detection of diverse impairment patterns; (ii) minimize total administration time to reduce participant burden, fatigue effects, and study costs; (iii) align with specific research objectives; and (iv) consider target population characteristics such as educational level, sensory abilities, and physical capabilities that may affect test feasibility.

The evidence reviewed supports prioritizing neuropsychological instruments with superior sensitivity for detecting MCI and early-stage dementia over traditional instruments that may miss subtle impairment. Specifically, the MoCA warrants consideration as the primary global screening instrument for Spanish older populations. Since validation evidence continues to accumulate, future research directly comparing MoCA and MMSE in diverse Spanish populations—including low-education groups and different cultural groups—would provide additional evidence supporting this recommendation.

In conclusion, current Spanish research practice emphasizes practical feasibility (brief screening) over comprehensive domain assessment, potentially limiting detection of early cognitive changes in specific domains. Strategic test combination could enhance comprehensiveness while remaining feasible for clinical and research implementation in Spanish older adult populations. This scoping review provides an evidence-based foundation for selecting appropriate cognitive assessment tools, supporting early detection of cognitive impairment, and informing the development of standardized, culturally appropriate neuropsychological batteries.

## 6. Limitations

This scoping review has several important limitations that should be considered when interpreting findings and recommendations.

The reviewed studies provide limited stratification by educational level, and normative data for low-education Spanish older adults remain substantially underdeveloped.

The restriction to research conducted in Spain and to the Spanish population, while justified by study aims, limits generalizability to broader international cognitive neuroscience literature and may miss relevant methodological innovations. Nevertheless, since neuropsychological tests are susceptible to cultural influences, this review offers invaluable insights into the conduct of cognitive assessments in Spanish samples.

As a scoping review, this study did not perform formal methodological quality appraisal or synthesis of diagnostic-accuracy metrics. Consequently, conclusions relate to patterns of instrument use rather than strength of evidence or comparative performance.

The 10-year review period (2013–2023) provides valuable temporal scope but may not fully capture accelerating methodological shifts occurring in cognitive assessment. While MoCA appeared in only 6.4% of the 47 reviewed studies, preliminary observation suggests that MoCA adoption has substantially increased in recent publications (outside the review period). Furthermore, the review did not systematically examine temporal trends in test selection across the decade, which might reveal evolving preferences and identify factors driving methodological changes.

Finally, the selection of studies in Spanish and English may have led to omission of some studies but is unlikely to have significantly affected the identification of core instruments used in Spanish older adults, as these languages cover the vast majority of cognitive screening conducted in Spain.

## Figures and Tables

**Table 1:** Characteristics of the selected studies.

Reference	Placement	Objectives	Study Design	Characteristics of the Sample				Measurement of CI	Covariates	Outcomes
				N	Age Mean (SD)	Sex % (n)	Other Information			
Recio-Rodríguez et al., 2022 [16].	Three healthcare centers in Salamanca and Valladolid.	Assess the efficacy of the combined use of smartphone and smartband technology for 3 months alongside brief lifestyle counseling, versus counseling alone, increasing physical activity, dietary habits, body composition, quality of life, level of functionality and cognitive performance.	RCT (NCT03574480)	160	65–80 70.8 (4)	M: 38.7% (62) F: 61.3% (98)	Cognitively healthy	MMSE, CDT, SVF-animals	Age, gender, marital status, cohabitation, dietary habits, physical activity, comorbidities, anthropometry, smoking habit, obesity, drug treatments, Pfeffer’s FAQ, QoL, Mediterranean diet adherence, physical activity.	The combined use of a smartphone app and smartband for 3 months did not result in lifestyle changes related to the amount of physical activity or eating habits and other clinical parameters compared to brief lifestyle advice. Only differences were reported at the cognitive level, where a slight improvement was observed in the CDT score.
Contador et al., 2018 [17].	Multicentre. Different socioeconomic areas in Madrid.	Investigate the existence of different patterns of functional impairment in older adults based on Pfeffer’s FAQ.	Cohort	3873	>65 73.6 (6.4)	M: 43.5% (1690) F: 56.5% (2188)	Without dementia	MMSE	Age, sex, educational attainment, living area, comorbidity index, self-perception of health.	The response patterns revealed the presence of three latent classes: (1) absence of alteration; (2) established functional alteration; and (3) minimal functional alteration.
Calatayud et al., 2021 [18].	Primary Healthcare center in Zaragoza.	Examine gender differences in cognitive performance in older adults with SMC.	Cross-sectional	367	≥65 M: 73.85 (5.99)	M: 33.5% (123) F: 66.5% (244)	SMC	MMSE and set test (SVF- 4 categories)	Age, gender, level of education, civil status, mental occupational status, physical occupational and clinical states, level of education.	Cognitive attention/calculation domains values were higher for men. Verbal fluency was higher for women but not statistically significant. Further research is needed.
Climent Catalá et al., 2018 [19].	Fourteen community pharmacies in Comunidad Valenciana.	Identify risk factors in lifestyle associated with the development of CI.	Cross-sectional	729	>65 M: 74.4 (6.4)	M: 40.1% (292) F: 59.9% (437)	SMC	MMSE and SPMSQ	Age, gender, educational level, physical exercise, sleep hours.	A total of 17.6% of the participants presented test scores compatible with CI. It was found that sleeping more than 9 h was associated with the development of CI. Exercise and poor sleeping hours were not associated with CI. Changes in sleep patterns and increasing the hours of sleep, may be a warning signal for a possible development of CI.
Gomez-Soria et al., 2020 [20].	San José Norte- Centro Healthcare Center in Zaragoza.	Evaluate the impact of a cognitive stimulation program in MCI at the cognitive level on ADL, and levels of anxiety and depression.	RCT (NCT03831061)	122	>65 M:75.01 (6)	M: 23% (28) F: 77% (94)	Cognitively healthy, score >60 in Barthel Index	MMSE	Age, sex, marital status, educational level, Barthel Index, Lawton and Brody Scale, Goldberg Questionnaire (anxiety sub-scale) and the Yesavage Geriatric Depression Scale (GDS-15).	The intervention group showed a significant improvement in cognitive function at both timepoints. The findings showed cognitive improvements in an older population with MCI in the short and medium term and improved basic ADLs in the short term.
Bisbe et al., 2020 [21].	Diagnostic Unit of Fundació ACE, Institut Català de Neurociències Aplicades, Barcelona.	Compare the cognitive effects of choreographed exercise with a multimodal physical therapy program in older adults with amnestic MCI.	RCT	31	65-85 M: 74.87 (5.38)	M: 51.62% (16) F: 48.38% (15)	Amnestic MCI	MMSE, Word list learning test and visual memory subtest (WMS-III), TMT, VFT, BNT, JLOT	Age, gender, education years, ADL, psycho-affective symptoms, quality of life, physical functioning.	Both groups significantly improved in visual delayed recall. The Choreography group exhibited significantly more benefits in verbal recognition memory than the Physical Therapy group.
Formiga et al., 2014 [22].	Seven primary care centers in Barcelona.	Examine the incidence of functional or cognitive impairment and its associated factors in a sample of individuals aged 85 years or older with and without diabetes mellitus, who were free of significant impairment at baseline.	Prospective	167	85 + 2-year follow-up *	M: 39.52% (66) F: 60.47% (101)	Cognitively healthy; diabetes vs. no diabetes	MMSE	Gender, marital status, education level, visual or auditory disabilities, IADL, MNA, Charlson Comorbidity Index, chronic diseases, and drug prescriptions.	In the oldest group, community-dwelling individuals without evidence of severe functional impairment at baseline, diabetes increases the risk of incident disability in only 2 years.
Contador et al., 2019 [23].	Multicentred. Different socioeconomic areas in Madrid.	Investigate the mortality rates of three types of disability and their specific explanatory factors in older adults.	Cohort	3816	>65 M: 73.56 (6.43)	M: 43.3% (1652) F: 56.7% (2164)	Without dementia	MMSE	Age, sex, level of education, comorbidity index, alcohol consumption, smoking habits, depression, living arrangement, living area.	FAQ and self-perceived functional limitations were associated with a higher risk of mortality at 5 years.
Gómez-Soria et al., 2021 [24].	San José Norte-Centro Healthcare Center, Zaragoza.	Analyze the long-term effects of a personalized cognitive stimulation program on the global cognition, cognitive aspects, IADL, anxiety, and depression in older adults with possible MCI.	RCT	50	≥65 M: 74.32 (5.47)	M: 22% (11) F: 78% (39)	Possible MCI (24–27 in MMSE)	MMSE	Age, sex, marital status, educational level, hypertension, diabetes, HC, obesity, stroke, visual and hearing impairment.	There were significant differences between the groups after 12 months in global cognition, global and spatial orientation, favoring the intervention group.
Gómez-Soria et al., 2023 [25].	Primary Healthcare center in the city of Zaragoza.	Evaluate the effects of a personalized-adapted cognitive stimulation program in older adults on global cognition, neuropsychological constructs, IADL, and mood.	RCT	337	≥65 M: 74 (6)	M: 30.27% (102) F: 69.73% (235)	Patients classified into 4 groups: no deterioration, SMC, level deterioration, moderate deterioration	MMSE	Age, gender, civil status, education level, physical and mental occupational status, nucleus of family coexistence, clinical characteristics, physical activity, smoking habits, environmental variables.	The intervention showed a tendency for improvement on global cognition and different cognitive functions for groups with no deterioration or level deterioration. The group with moderate deterioration improved in anxiety.
Calatayud et al., 2023 [26].	Primary Healthcare center in the city of Zaragoza.	Address the effectiveness of language stimulation programs on cognitive levels in older adults.	RCT	308	≥65 M: 73.66 (5.88)	M: 35.1% (108) F: 64.9% (200)	With and without SMC	MMSE and set test (SVF-4 categories)	Age, gender, civil status, education level, physical and mental occupational status, HBP, diabetes, hypercholesterolemia, obesity and cerebrovascular accident.	The comprehensive cognitive stimulation program has made it possible to improve the global aspects of cognition, language proficiency, and verbal fluency.
Mora et al., 2013 [27].	Mataró Aging Study.	Study obestatin concentrations in relation to handgrip strength, functional capacity and cognitive state in older women.	Prospective	110	69–101 M: 76.93 (6.32)	F: 100% (110)	All participants were women	MMSE	Age, comorbidities, falls, hours walking, frailty, independence, GDS.	Higher obestatin levels were associated with increased weakness. Obestatin is associated with low muscle strength and impaired functional and cognitive capacity in older women.
García-Esquinas et al., 2022 [28].	ENRICA-2 cohort.	Examine the association of serum cotinine (as a measure of second-hand smoke exposure) and cognitive function in older adults.	Cohort	2087	≥65 No mean age available	M: 47.39% (989) F: 52.61% (1098)	Disability-free older adults	MMSE, DS backwards, TMT, FCSRT, SVF	Sex, age, education level, cohabitation, physical activity, anthropometric measures, comorbidities, ADL.	An increased risk of global cognitive impairment and decline in working memory performances was observed in older adults exposed to second-hand smoke.
Díaz Navarro et al., 2019 [29].	Nine Basic Health Zones in La Palma, The Canary Islands, Spain.	Determine the prevalence and profile of frailty in the island of La Palma, The Canary Islands, Spain.	Cross-sectional	592	>70 M: 79 (6)	F: 61% (361) M: 39% (231)	Community-dwelling older adults	MMSE	Age, sex, marital status, education, cohabitation, anthropometry, nutritional status, physical activity, comorbidities, polypharmacy, clinical history.	The prevalence of frailty in people over 70 years was estimated at 20%.
Formiga et al., 2014 [30].	OCTABAIX study.	Evaluate whether thyroid status in older subjects correlates with physical and cognitive function at baseline and with 3-year mortality.	Cohort	307	85 years at baseline	F: 54.6% (184) M: 45.4% (123)	All participants were born in 1924 (85 years old at baseline)	MMSE	Gender, marital status, education level, successful aging, ADL, QoL, falls, comorbidities and polypharmacy.	There was no association of TSH or thyroid disorders with physical or cognitive function.

ADL: Activities of daily living, BNT: Boston Naming Test, CDT: Clock Drawing Test, CI: cognitive impairment, DS: Digit Span, FAQ: Functional Activities Questionnaire, FCSRT: Free and Cued Selective Reminding Test, GDS: Geriatric Depression Scale, HBP: High blood pressure, JLOT: Judgment of Line Orientation Test, MCI: mild cognitive impairment, MMSE: Mini-Mental State Examination, MNA: Mini Nutritional Assessment, PVF: Phonetic verbal fluency, QoL: quality of life, RCT: Randomized controlled trial, SMC: subjective memory complaints, SPMSQ: Short Portable Mental State Questionnaire, SVF: semantic verbal fluency, TMT: Trail Making Test, VFT: verbal fluency tests (SVF and PVF), WMS-III: Weschler Memory Scale, Third Edition. * All participants were born in 1924 (85 at baseline).

**Table 2:** Characteristics of the most used neuropsychological tests.

Neuropsychological Test	No. of Articles in the Review That Use It	Description	Administration Time	Cognitive Domains Assessed	Administration Method	Target	Limitations
Mini-Mental State Examination (MMSE)	41	General cognition paper-and-pencil test that assesses multiple cognitive domains through different tasks.	10 min.	Attention, language, memory and learning, perceptual-motor.	In person/Telephonic adaptation: STICS-m.	Broad field of target populations in clinical, research and community settings.	Results can be influenced by patients age, educational level, emotional status or personality. Items are insufficient to detect the change from MCI to advanced CI. Administration takes longer than other tests.
Verbal Fluency Tests—Semantic (SVF) and Phonetic (PVF)	18	Patients are asked to say as many words as they can within a category (SVF) or beginning with a certain letter (PVF) in 1 min.	1 min per category (SVF) or letter (PVF).	Language, memory.	In person.	MCI and incident dementia patients.	VF is also affected in brain damage, stroke, aphasia, cortical dementias and other degenerative diseases.
Trail Making Test (TMT)	12	Patients are asked to connect, in order and as quicky as possible, randomly arranged circles containing numbers and letters.	Maximum time of 300 s.	Perceptual-motor, memory and learning, attention, executive function.	In person.	Brain dysfunction indicator, distinguish neurological patients from healthy elders.	Results can be affected by age and educational level.
Digit Span (DS)	11	Patients are asked to repeat a series of numbers, forwards and backwards.	1–3 min	Executive function, attention, memory and learning.	In person.	Widely used in neuropsychological research and clinical evaluation of all ages.	Results can be affected by age, education and culture. Results can also be impaired in traumatic brain injury and chronic fatigue syndrome.
Clock Drawing Test (CDT)	6	Patients are asked to draw a clock with hands, with all the numbers and marking a specific time.	Less than 5 min.	Executive function, memory and learning, attention, perceptual-motor.	In person.	Screening of CI, AD, Parkinson Disease and other dementias in elders.	Results can be affected by age and education.
Boston Naming Test (BNT)	6	Patients are asked to identify items shown in different images.	Maximum time 20 min (20 s per image).	Language, memory and learning.	In person.	All age groups, from young children to elders.	Results can be affected by age, education, living environment and other sociodemographic variables. Administration time may be long in patients with low scores.

Summary of the six most frequently used neuropsychological tests identified in this scoping review. For each test, the table presents frequency of use in reviewed studies (number and percentage), brief description, administration method, approximate administration time (minutes), cognitive domains assessed according to DSM-5 framework, appropriate target populations, and key limitations for clinical and research interpretation. AD: Alzheimer’s disease, BNT: Boston Naming Test, CDT: Clock Drawing Test, CI: cognitive impairment, DS: Digit Span, JLOT: Judgment of Line Orientation Test, MCI: mild cognitive impairment, MMSE: Mini-Mental State Examination, PVF: Phonetic verbal fluency, SMC: subjective memory complaints, SPMSQ: Short Portable Mental State Questionnaire, SVF: semantic verbal fluency, TMT: Trail Making Test, VF: verbal fluency, VFT: verbal fluency tests (SVF and PVF).

## Data Availability

No new data were created or analyzed in this study. Data sharing is not applicable to this article.

## Supplementary Materials

The following supporting information can be downloaded at https://www.mdpi.com/article/10.3390/geriatrics11020045/s1; File S1: Search Strategy; Table S1: Table 1 complete. References [51,52,53,54,55,56,57,58,59,60,61,62,63,64,65,66,67,68,69,70,71,72,73,74,75,76,77,78,79,80,81,82] are cited in the supplementary materials.

## Abbreviations

The following abbreviations are used in this manuscript:

AD	Alzheimer’s Disease
BNT	Boston Naming Test
CDT	Clock Drawing Test
CI	Cognitive Impairment
DS	Digit Span
DSM-5	Diagnostic and Statistical Manual, Fifth Edition
JLOT	Judgment of Line Orientation Test
MCI	Mild Cognitive Impairment
MMSE	Mini-Mental State Examination
MoCA	Montreal Cognitive Assessment
PVF	Phonemic Verbal Fluency
RCT	Randomized Controlled Trial
SMC	Subjective Memory Complaints
SVF	Semantic Verbal Fluency
TMT	Trail Making Test
VF	Verbal Fluency
VFT	Verbal Fluency Test
WMS-III	Weschler Memory Scale, Third Edition
