# Supplementary material for: Cognitive Testing in Spanish Older Adults: A Scoping Review
Source: Geriatrics (Basel). 2026 Apr 10;11(2):45. doi: 10.3390/geriatrics11020045 (PMC13116379; doi:10.3390/geriatrics11020045)
Supplement: Supplementary file 1 [file geriatrics-11-00045-s001.zip › SM1 Search Strategy.pdf]

Supplementary material: Search Strategy

1. neuropsychol*
2. cognit*
3. neurocognit*
4. mental
5. brain
6. #1 OR #2 OR # 3 OR #4 OR #5
7. assessment
8. test*
9. "screening tool"
10. Questionnaire
11. #7 OR #8 OR #9 OR #10
12. Alzheimer
13. "cognitive impairment"
14. "cognitive decline"
15. "cognitive function"
16. Dementia
17. #12 OR #13 OR #14 OR #15 OR #16
18. #6 AND #11 AND #17
19. Spanish
20. #18 AND #19
