# Supplementary material for: Cognitive Testing in Spanish Older Adults: A Scoping Review
Source: Geriatrics (Basel). 2026 Apr 10;11(2):45. doi: 10.3390/geriatrics11020045 (PMC13116379; doi:10.3390/geriatrics11020045)
Supplement: Supplementary file 1 [file geriatrics-11-00045-s001.zip › SM1 Table 1 corrected version.pdf]

## Supplementary material: Table 1 Complete

**Table1. Characteristics of the selected studies**

| Reference | Placement | Objectives | Study design | Characteristics of the sample | | | | Measurement of CI | Covariates | Outcomes |
|---|---|---|---|---|---|---|---|---|---|---|
| | | | | **N** | **Age** Mean (SD) | **Sex** % (n) | **Other information** | | | |
| (Alegret et al., 2018 [51]) | Memory Clinic of Fundació ACE, *Institut Català de Neurociències Aplicades* (Barcelona, Spain) | Investigate the presence of verbal fluency déficits in MCI and AD | Prospective | 1820 | >44<br>M: 70.55 (8.1) | F: 62.75% (1142)<br>M: 37.25% (678) | 568 CH, 885 MCI, and 367 mild AD | VF, NBACE battery | Age, sex, education years | Lower performances on VF were significantly related to conversion from CH to MCI/MCI to dementia |
| (Alegret et al., 2015 [52]) | Memory Clinic of Fundació ACE, Institut Català de Neurociències Aplicades, Barcelona. | Determine whether higher subjective memory impairment (SMI) was related to worse memory performance (S-FNAME) or associated with greater affective symptoms whether MFE-30 and FNAME were able to discriminate between cognitively normal and MCI subjects. | Cross-sectional | 317 | >49<br>63.79 (7.75) | M: 29% (92)<br>F: 71% (225) | Cognitively normal and MCI | MMSE, FNAME, MFE-30.<br>Temporal, Spatial and Personal Orientation; DS, Block Design and Similarities subtests of WAIS-III; The Word List Learning test from the WMS-III, Verbal comprehension; an abbreviated 15-item BNT; the Poppelreuter test; CDT; Ideomotor and Imitation praxis; the Automatic Inhibition subtest of the Syndrom Kurtz Test; PVF-letter p, SVF-animals, 15-Objects test. | Age, gender, education years | SMI was more related to depressive symptoms than to S-FNAME scores.<br>S- FNAME scores were related to other episodic memory test performances, but neither to affective symptoms nor to SMI.<br>MFE-30 and S-FNAME are not optimal for discriminating between CN and MCI groups. |
| (Bayes-Marin et al., 2020 [53]) | Data form "Edad con Salud" Study | Identify distinct groups of immediate and delayed verbal memory in two age subsamples, and to analyze associated factors. | Prospective | 1089 | >50<br>M: 63.48 (No SD Available) | F: 53.07% (578)<br>M: 46.93% (511) | Stratified by age, 50-65 and >65 | Immediate and delayed verbal recall and MMSE | Age, sex, marital stuts, level of education, occupation, urban/rural, comorbidities, smoking habits, alcohol consumption, physical activity, disability, QoL. | Low initial performance and decline were associated with older age, lower education, and higher diabetes/stroke prevalence. |

| | | | | | | | | | | |
|---|---|---|---|---|---|---|---|---|---|---|
| (Bisbe et al., 2020 [21]) | Diagnostic Unit of Fundació ACE, Institut Català de Neurociències Aplicades, Barcelona. | Compare the cognitive effects of choreographed exercise with a multimodal physical therapy program in older adults with amnestic MCI. | RCT | 31 | 65-85<br>M: 74.87 (5.38) | M: 51.62% (16)<br>F: 48.38% (15) | Amnestic MCI | MMSE, Word list learning test (WMS-III), visual memory subtest (Repeatable for the Assessment of Neuropsychological status), TMT, PVF, SVF, BNT and JLOT. | Age, gender, education years, ADL, psycho-affective symptoms, quality of life, physical functioning. | Both groups significantly improved in visual delayed recall. The Choreography group exhibited significantly more benefits on verbal recognition memory than the Physical Therapy group. |
| (Calatayud et al., 2021 [18]) | Primary Healthcare centre in Zaragoza | Examine gender differences in cognitive performance in older adults with subjective memory complaints (SMC) | Cross-sectional | 367 | ≥65<br>M: 73.85 (5.99) | M: 33.5% (123)<br>F: 66.5% (244) | SMC | MMSE and SVF (set test) | Age, gender, level of education, civil status, mental occupational, physical occupational and clinical states (HBP, diabetes, cholesterol, obesity, CVA). | Cognitive attention/calculation domains values were higher for men. Verbal fluency was higher for women but not statistically significant. Further research is needed. |
| (Calatayud et al., 2023 [26]) | Primary care center in the city of Zaragoza (northeastern Spain) | Address the effectiveness of language stimulation programs by cognitive levels in elders. | RCT | 308 | ≥65<br>M: 73.66 (5.88) | M: 35.1% (108)<br>F: 64.9% (200) | With and without SMC | MMSE and SVF (set-test 4 categories) | Age, gender, civil status, education level, physical and mental occupational status, HBP, diabetes, hypercholesterolemia, obesity and cerebrovascular accident. | The comprehensive cognitive stimulation program has made it possible to improve the global aspects of cognition, language proficiency, and verbal fluency. |
| (Climent et al., 2015 [54]) | 14 community pharmacies in Comunidad Valenciana | Identify risk factors in lifestyle associated with the development of CI. | Cross-sectional | 729 | >65<br>M: 74.4 (6.4) | M: 40.1% (292)<br>F: 59.9% (437) | SMC | MMSE and SPMSQ | Age, gender, educational level, physical exercise, sleep hours. | 17.6% of the participants presented test scores compatible with CI. It was found that sleeping more than 9 hours was associated with the development of CI. Exercise and poor sleeping hours were not associated with CI. Changes in sleep patterns, increasing the hours of sleep, may be a warning signal for a possible development of CI. |
| (Contador et al., 2019 [23]) | Multicentred. Different socioeconomic areas in Madrid. | Investigate the mortality rates of three types of disability and their specific explanatory factors in older adults. | Cohort | 3816 | >65<br>M: 73.56 (6.43) | M: 43.3% (1652)<br>F: 56.7% (2164) | Without dementia | MMSE | Age, sex, level of education, comorbidity index, alcohol consumption, smoking habits, depression, living arrangement, living area. | FAQ and self-perceived functional limitations were associated with a higher risk of mortality at 5-years. |
| (Contador et al., 2018 [17]) | Multicentred. Different | Investigates the existence of different patterns of | Cohort | 3873 | >65<br>73.6 (6.4) | M: 43.5% (1690) | Without dementia | MMSE | Age, sex, educational attainment, living area, | The response patters revealed the presence of three latent classes: |

| | | | | | | | | | | |
|---|---|---|---|---|---|---|---|---|---|---|
| | socioeconomic areas in Madrid. | functional impairment in older adults based on Pfeffer's FAQ | | | | F: 56.5% (2188) | | | comorbidity index, self-perception of health. | 1) absence of alteration; 2) established functional alteration; and 3) minimal functional alteration. |
| (Delgado-Lima et al., 2023 [55]) | Geriatric Department from Hospital Central de la Cruz Roja "San José y Santa Adela", Madrid. | Elucidate the associations between cognitive status and olfactory identification performance in aging; understand the predictive value of olfactory capacity in identifying CI risk and study how both CI and olfactory capacity relate to other wellness in aging variables. | Cross-sectional | 149 | 60-90 M: 77.15 (7.29) | M: 51.01% (76) F: 48.99% (73) | No prior diagnosis of dementia. Patients divided into: Cognitively healthy, MCI and Severe CI. | MoCA | Age, sex, previous COVID-19 diagnosis, allergies, smoking habits, alcohol consumption, ADL, Vitamins B12, D, Albumin, Cholesterol, Lymphocytes | The predictive ability of olfactory identification scores for the risk of mild and severe impairment is around 80%. Olfactory identification decreases with cognitive function. Performance in odour identification is associated with impairment of episodic memory and executive functions. |
| (Díaz Navarro et al., 2019 [29]) | 9 Basic Health Zones in La Palma, The Canary Islands, Spain. | Determine the prevalence and profile of frailty in the island of La Palma, The Canary Islands, Spain. | Cross-sectional | 592 | >70 M: 79 (6) | F: 61% (361) M: 39% (231) | Community dwelling older adults | MMSE | Age, sex, marital status, education level, cohabitation, anthropometry, nutritional status, physical activity, comorbidities, polypharmacy and clinical history. | The prevalence of frailty in people over 70 years was estimated at 20%. |
| (Fernández-Matarrubia et al., 2021 [56]) | Universidad de Navarra, Spain | Assess the association between an active lifestyle score and leisure-time physical activity and changes in cognitive function | Cohort | 806 | >55 M: 66 (5) | M: 69.7% (562) F: 30.3% (244) | Participants were all students from the University of Navarra. | STICS-m | Age, sex, follow-up time, years of university education, smoking habits, total energy intake, anthropometry, BMI, alcohol intake, comorbidities, dietary habits. | An active lifestyle is associated with a better status of cognitive function over time only among *APOE*-4 non-carriers. |
| (Ferreira et al., 2016 [57]) | GENIC database (Group of Neuropsychological Studies of the Canary Islands) University of La Laguna, and Hospital Universitario de Canarias, Tenerife. | Investigate different proxies of brain and cognitive reserve as potential mediators of the effect of cortical thinning on cognition in healthy middle-aged adults. | Cross-sectional | 82 | 40-50 M: 45.1 (3.9) | M: 49% (40) F: 51% (42) | Healthy | MMSE, Block design from WAIS-III, JLOT, Color Trail Test, CVLT. | Age, gender, years of education, cognitive reserve, total intracranial volume. | Higher reserve buffers the effect of cortical thinning on cognition in healthy middle-aged adults. |
| (Formiga et al., 2014[a] [30]) | Data from OCTABAIX study | Evaluate whether thyroid status in older subjects correlates with physical and cognitive function at baseline and with 3-year mortality. | Cohort | 307 | 85 years at baseline | F: 54.6% (184) M: 45.4% (123) | All participants were born in 1924 (85 years old at baseline) | MMSE | Gender, marital status, education level, successful ageing, ADL, QoL, falls, comorbidities and polypharmacy. | There was no association of TSH or thyroid disorders with physical or cognitive function. |

| | | | | | | | | | | |
|---|---|---|---|---|---|---|---|---|---|---|
| (Formiga et al., 2014b [22]) | 7 primary care centres in Barcelona. | Examine the incidence of functional or cognitive impairment and its associated factors in a sample of individuals aged 85 years or older with and without diabetes mellitus, who were free of significant impairment at baseline | Prospective | 167 | 85 + 2-year follow-up **All participants were born in 1924 (85 at baseline)* | M: 39.52% (66) F: 60.47% (101) | Cognitively healthy. Diabetes vs. No diabetes. | MMSE | Gender, marital status, education level, visual or auditory disabilities, ADL, MNA, Charlson's Comorbidity Index, chronic diseases, and drug prescriptions. | In the oldest old, community-dwelling individuals without evidence of severe functional impairment at baseline, diabetes increases the risk of incident disability in only 2 years. |
| (Galbete et al., 2015 [58]) | Universidad de Navarra | Evaluate the association between adherence to the mediterranean diet and cognitive function | Cohort | 823 | >55 M: 67.4 (5.7) | M: 71% (597) F: 29% (223) | Participants were all university students, cognitively healthy | STICS-m | Age, Sex, years of university education, BMI, physical activity, smoking habits, energy intake, total fat, proteins, and carbohydrates (% of energy) | Higher cognitive decline was observed among participants with lower adherence to mediterranean diet. |
| (García Villanueva et al., 2014 [59]) | Hospital Universitario de Basurto and Associations of retired persos of Bilbao, Basque Country, Spain | Analyse Quality of life (QoL) of persons over 60 years of age in Bizkaia, and the rela- tion and possible influence of the perception of the state of health with the cognitive level and the activities that they carry out. | Prospective | 317 | >60 M: 74.16 (No SD Available) | F: 74.4% (236) M: 25.6% (81) | Without cognitive impairment. | MMSE, Global deterioration scale, Text memory subtest of Barcelona Test. | Age, sex, education years, cohabitation, comorbidities, leisure activities. | Participants showed values in quality of life that exceded the reference values for Spanish population except in that of bodily pain. There were low correlations between QoL and mental, physical and social activities. |
| (García-Esquinas et al., 2022 [28]) | ENRICA-2 cohort | Examine the association of serum cotinine (as a measure of second-hand smoke exposure) and cognitive function in older adults. | Cohort | 2087 | ≥65 No mean age available | M: 47.39% (989) F: 52.61% (1098) | Disability free older adults | MMSE, DS, TMT, FCSRT, SVF | Sex, age, education level, cohabitation, physical activity, anthropometric measures, comorbidities, ADL. | An increased risk of global cognitive impairment and declines in working memory performances was observed in older adults exposed to second-hand smoke. |
| (García-Garro et al., 2020 [60]) | Two associations of postmenopausal women in Jaén. | Determine the effects of Pilates exercise program on the cognitive and physical functioning of older Spanish Women. | RCT | 110 | ≥60 M: 68.18 (8.35) | F: 100% (110) | Women | MMSE, TMT, SVF (Isaacs test: animals, colours, fruits and cities) | Age, height, weight, BMI, occupation, marital status, education level. | This study suggests that the women in the pilates group experienced improvements across all the variables except for global cognitive function. |
| (Gil-Peinado et al., 2023 [61]) | 28 Community Pharmacies, 1 Primary Care Health Centre, and 1 Hospital. | Estimate the relationship between risk and protective factors associated with dementia | Cross-sectional | 709 | >50 M: 69.3 (10.3) | F: 73.8% (523) M: 26.2% (186) | Patients with concerns about their cognition | MIS, SPMSQ, SVF | Age, sex and factors A-to-Z Dementia Knowledge List. | Prevalence of CI was 22.6%. Living alone, having diabetes, taking benzodiazepines, and sleeping more than 9 h were statistically significantly |

| | | | | | | | | | | |
|---|---|---|---|---|---|---|---|---|---|---|
| | | | | | | | | | | associated with CI, whereas doing memory training or a family history of dementia was characteristic of patients without CI. |
| (Gómez-Soria et al., 2021 [24]) | San José Norte-Centro Healthcare Centre, Zaragoza. | Analyse the long-term effects of a personalized cognitive stimulation program on the global cognition, cognitive aspects, IADL, anxiety, and depression in older adults with possible MCI. | RCT | 50 | ≥65<br>M: 74.32 (5.47) | M: 22% (11)<br>F: 78% (39) | Possible MCI (24-27 in MMSE) | MMSE | Age, sex, marital status, educational level, hypertension, diabetes, cholesterol, obesity, stroke, visual and hearing impairment. | There were significant differences between the groups after 12 months in global cognition, global and spatial orientation, in favour of the intervention group. |
| (Gomez-Soria et al., 2020 [20]) | San José Norte-Centro Healthcare Center in Zaragoza | Evaluate the impact of a cognitive stimulation program in MCI at the cognitive level on activities of daily living (ADLs), and levels of anxiety and depression. | RCT (NCT03831061) | 122 | >65<br>M:75.01 (6) | M: 23% (28)<br>F: 77% (94) | Cognitively Healthy, score >60 in Barthel Index | MMSE | Age, sex, marital status, educational level, ADL, Lawton and Brody Scale, Goldberg Questionnaire (anxiety sub-scale) and the Yesavage GDS-15 | The intervention group showed a significant improvement in cognitive function at both timepoints. The findings showed cognitive improvements in an older population with MCI in the short and medium-term and improved basic ADLs in the short term. |
| (Gómez-Soria et al., 2023 [25]) | Primary Healthcare centre in a city of northeastern Spain. | Evaluate the effects of a personalized-adapted cognitive stimulation program in older adults on global cognition, neuropsychological constructs, IADL, and mood. | RCT | 337 | ≥65<br>M: 74 (6) | M: 30.27% (102)<br>F: 69.73% (235) | Patients classified into 4 groups: No deterioration, SCI, level deterioration, moderate deterioration | MMSE | Age, gender, civil status, education level, physical and mental occupational status, nucleus of family coexistence, clinical characteristics, physical activity, smoking habits, environmental variables. | The intervention showed a tendency of improvement on global cognition and different cognitive functions for groups with no deterioration or level deterioration. The group with moderate deterioration improved in anxiety. |
| (Goni et al., 2020 [62]) | Universidad de Navarra, Spain | Assess the association between polyphenol intake and 6-year change in cognitive function | Cohort | 806 | >50<br>M: 60.7 (5.6) | F: 30.3%<br>M: 69.7% | Participants were all students from the University of Navarra, cognitively healthy | STICS-m | Age, sex, follow-up time, years of university education, APOE-E4, smoking habits, total energy intake, physical activity, weight, height, BMI, alcohol intake, comorbidities, dietary habits | No significative association between polyphenol intake and changes in cognitive function was found. The results suggest that lignan and stilbene intake was associated with improvements in cognitive function. |

批注 [NP1]: Please check if this should be “Goni et al.,2020”

| | | | | | | | | | | |
|---|---|---|---|---|---|---|---|---|---|---|
| | | | | | | | | | | particularly with respect to immediate memory and language domains. |
| (González et al., 2017 [63]) | Longitudinal Study Aging in Spain. “ELES Project” | Analyse the predictors of cognitive performance in a Spanish sample over 50 years from a multidimensional perspective, including socioeconomic, affective, and physical variables. | Cross-sectional | 832 | 50-89<br>M: 64.72 (10.27) | F: 49.1% (408)<br>M: 50.9% (424) | Cognitively healthy, stratified by age | AVLT, Letter cancellation task (ELSA Study), DS backwards, VFT, BNT. | Age, sex, education years, economic status, physical activity, subjective health, satisfaction with aging, grip strength, BMI. | Education years is the variable that best predicts cognitive performance until 79 years old. In the 80+ group, economic status is the best predictor. |
| (Lara et al., 2019 [40]) | Household survey around various regions of Spain | Investigate the effect of loneliness and social isolation on distinct cognitive domains over a 3-year follow-up period in a population-based sample of middle and older-aged adults. | Prospective | 1691 | >50<br>M: 64.5 (9.8) | M: 47.2% (798)<br>F: 52.8% (893) | | MMSE and 5 performance tests: Immediate and delayed verbal recall, WAIS-III DS forward and backwards, SVF -animals | Age, sex, years of education, physical activity, alcohol consumption, disability, depression, history of stroke and diabetes. | Loneliness and social isolation are associated with decreased cognitive function over a 3-year follow-up period |
| (Lara et al., 2015 [64]) | Computer-Assisted Personal Interviewing to a Spanish sample of the Collaborative Research on Ageing in Europe project (COURAGE). | Examine how cognitive function may influence suicidal thoughts. | Cross-sectional | 4583 | ≥18<br>M: 47.64 (17.8) | M: 49.4% (2078)<br>F: 50.6% (2505) | Healthy population and with depression | MMSE, Word list immediate and delayed verbal recall, DS WAIS-III, SVF-animals | Age, gender, education level, marital status, depression, | Cognitive functioning and diagnosis of depression are associated with higher risk of suicide in the Spanish general population, especially in young individuals. |
| (Lojo-Seoane et al., 2019 [65]) | Primary healthcare centre in Santiago de Compostela | Study the influence of cognitive reserve (CR) on cognitive performance of individuals with SCC within a period of 36 months | Prospective | 212 | >50<br>M: 65.86 (8.89) | M: 31.6% (67)<br>F: 68.4 % (154) | SCC | MMSE, CAMCOG-R, CVLT, Counting and listening span tasks, Peabody picture-vocabulary test, WAIS-III vocabulary test | Age, gender, years of education, occupational attainment, reading habits, social activities. | This study confirmed the direct effects of CR in working memory and general cognition performance at baseline as well as indirect effects on episodic memory and working memory at follow-up. |
| (López et al., 2021 [66]) | Multicentred. PREDIMED-Plus Study | Evaluate the association between peripheral arterial disease (PAD) measured with the Ankle-brachial index (ABI) and cognitive performance in | Cross-sectional | 4898 | 55-75<br>M: 64.85 (4.95) | M:52.1% (2550)<br>F: 47.9% (2348) | Overweight or obesity and metabolic syndrome. | MMSE, SVF, PVF, WAIS-III working memory index, TMT, CDT. | Age, gender, years of education, blood pressure, smoking habits, diabetes, dyslipidaemia, anthropometric measures (height, weight, waist circumference, BMI), use of medication and family history of disease. | No association between ABI and cognitive performance was observed. |

| | | | | | | | | | | |
|---|---|---|---|---|---|---|---|---|---|---|
| | | individuals with overweight or obesity and metabolic syndrome. | | | | | | | | |
| (López-Higes et al., 2018 [67]) | Center for the Prevention of Cognitive Impairment, Madrid | Explore if cognitive reserve, executive functions, and working memory capacity are predictive of performance in the language domain after a cognitive training intervention. | Prospective | 66 | 60-80<br>M: 71.15 (6.6) | M: 67% (22)<br>F: 33% (44) | Cognitively healthy and SCD | MMSE, Stroop test, TMT, BNT. | Age, gender, years of education, GDS-15. | The SCD group presented greater benefits in the language domain than cognitively intact participants. |
| (Mora et al., 2013 [27]) | Mataró Ageing Study | Study obestatin concentrations in relation to handgrip strength, functional capacity and cognitive state in old women | Prospective | 110 | 69-101<br>M: 76.93 (6.32) | F: 100% (110) | All participants were women | MMSE | Age, comorbidities, falls, hours walking, frailty, independence, GDS. | Higher obestatin levels were associated to increased weakness. Obestatin is associated to low muscle strength and impaired functional and cognitive capacity in old women. |
| (Muñoz-Garach et al., 2021 [68]) | Data were extracted from the PREDIMED-Plus trial (23 Spanish centres) | Examine the association between milk and dairy products intake and the prevalence of cognitive decline among individuals at high cardiovascular risk. | Cross-sectional | 6426 | 55-75<br>M: 65 (4.9) | M: 51.5% (3309)<br>F: 48.5% (3117) | Overweight, obesity and metabolic syndrome | MMSE | Age, gender, educational level, employment status, smoking habits, comorbidities, medication use, anthropometry, and HBP. | A higher prevalence of cognitive decline was found in subjects who consumed more milk and dairy products. However, a positive correlation was found between the consumption of whole milk and the MMSE score. |
| (Muñoz-García et al., 2019 [69]) | Universidad de Navarra, Spain | Investigate associations between 5 *a priori* defined “high-quality” dietary patterns and changes in cognitive function. | Cohort | 806 | >55<br>M: 61.7 (6) | M: 69.7% (562)<br>F: 30.3% (244) | Participants were all students from the University of Navarra, cognitively healthy | STICS-m | Age, sex, follow-up time, years of university education, APOE-E4, smoking habits, total energy intake, physical activity, anthropometry, BMI, alcohol intake, comorbidities, dietary habits | A beneficial association between MIND diet and cognitive function was observed. Results also suggested a benefit in cognitive function for the AHEI-2010 pattern. |
| (Muñoz-García et al., 2019 [70]) | Universidad de Navarra, Spain | Assess the association between the consumption of sugar-swetened beverages or artificially swetened beverages and cognitive function. | Cohort | 806 | >55<br>M: 61 (6) | F: 30.3% (244)<br>M: 69.7% (562) | Cognitively healthy | STICs-m | Age, sex, APOE-E4 genotype, years of university education, comorbidities, smoking habits, BMI, physical activity, adherence to Mediterranean diet and total energy intake. | A significant association between the consumption of sugar-swetened beverages and changes in cognitive function was observed in the total sample. |
| (Muñoz-García et al., 2021 [71]) | Universidad de Navarra | Address the association between exploratory | Cohort | 806 | >55<br>M: 67 (5) | M: 70% (564) | Without SCI. Participants were all | STICS-m | Age, gender, years of university education, APOE 4, total energy intake, alcohol | Two dietary patterns were identified, |

| | | | | | | | | | | |
|---|---|---|---|---|---|---|---|---|---|---|
| | | (empirically derived) dietary patterns and changes in the STICS-m over 6 years. | | | | F: 30% (242) | students from the University of Navarra, cognitively healthy | | consumption, smoking habits, BMI, physical activity, comorbidities. | Western and Mediterranean. Adherence to Western dietary pattern was significantly associated with negative STICS-m changes after 6 years (greater CD), while Mediterranean diet was associated with less decline in cognitive function, thus it could help to lower dementia incidence |
| (Ni et al., 2022 [72]) | Data from the PREDIMED-Plus trial were analysed. | Assess the association between diary consumption and cognitive changes in old population with high cardiovascular risk. | Cohort | 4668 | 55-75<br>M: 65.0 (4.9) | M: 51.9% (2425)<br>F: 48.1% (2243) | High cardiovascular disease risk. | MMSE, SVF, PVF, DS, CDT and TMT. | Age, sex, education level, civil status, BMI, waist circumference, lifestyle variables (Smoking habits, physical activity…), medication use and dietary variables (food frequency consumption) | No clear prospective associations between consumption of most consumed dairy products and cognition were observed. However, there may be an association with a greater rate of CD over a 2-year period in older adults at high cardiovascular disease risk for whole-fat milk. |
| (Nishi et al., 2021 [73]) | Data were extracted from the PREDIMED-Plus trial (23 Spanish centres) | Examine the relationship between baseline adherence to three *a priori* dietary patterns (Mediterranean, DASH, and MIND diets) with 2-year changes in cognitive performance in older adults with overweight or obesity and high cardiovascular disease risk. | Cohort | 6647 | 55-75<br>M: 65 (4.9) | M: 52% (3456)<br>F: 48% (3191) | Overweight, obesity and metabolic syndrome | MMSE, TMT, CDT, VF (semantic and phonetic), DS (forward and backward) | Age, sex, education level, civil status, BMI, energy intake. smoking habits, alcohol consumption, comorbidities. | Higher baseline adherence to the Mediterranean dietary pattern may be associated with better cognitive performance than lower adherence over a period of 2 years. |
| (Nishi et al., 2023 [74]) | Data from the PREDIMED-Plus Study. (23 centers across Spain) | Longitudinally assess the association between hydration status, and water intake, with changes in cognition in an older Spanish population at high cardiovascular disease risk. | Cohort | 1957 | 55-75<br>M: 65 (4.9) | F: 50.5% (989)<br>M: 49.5% (968) | Overweight and metabolic syndrome | MMSE, VFT, DS, CDT and TMT | Age, sex education level, civil status, BMI, physical activity, smoking habits, sleeping hours, comorbidities, biochemical parameters, Mediterranean diet and water intake | Lower physiological hydration status was associated with a greater decline in global cognitive function. |

| | | | | | | | | | | |
|---|---|---|---|---|---|---|---|---|---|---|
| (Ramos et al., 2022 [75]) | 19 Community pharmacies in Comunidad Valenciana. | Investigate the relationship between anticholinergic burden and CI in adult Spanish subjects with SMC. | Cross-sectional | 512 | >50<br>M: 70.19 (8.50) | M: 26.17% (134)<br>F: 73.83% (378) | SMC | MIS, SPMSQ, SVF-animals | Age, gender, BMI, drugs, diabetes, hypertension, hypercholesterolemia, depression. | An association between CI and the anticholinergic burden was observed. |
| (Razquin et al., 2020 [76]) | Universidad de Navarra | Investigate the association between hypertension and cognitive function and to assess whether better adherence to the Mediterranean diet may modify this association. | Prospective | 764 | >55<br>M: 60.75 (5.6) | M: 18.6% (142)<br>F: 81.4% (622) | Participants were all students from the University of Navarra, cognitively healthy | STICS-m | Age, sex, physical activity, APOE-E4, diabetes, previous CVA, BMI, overweight, obesity, smoking habit, university education, total energy intake. | Hypertension was inversely associated with cognitive function, but an attenuation of this detrimental association by a moderate/high adherence to the Mediterranean diet was suggested. |
| (Recio-Rodríguez et al., 2022 [16]) | 3 health care centres in Salamanca and Valladolid | Assess the efficacy of the combined use of smartphone and smartband technology for 3 months alongside brief lifestyle counselling, versus counselling alone, in increasing physical activity., dietary habits, body composition, quality of life, level of functionality and cognitive performance. | RCT (NCT03574480) | 160 | 65-80<br>70.8 (4) | M:38.7% (62)<br>F: 61.3% (98) | Cognitively healthy | MMSE, CDT, SVF-animals | Age, gender, marital status, number of cohabitants, dietary habits state of change, physical activity state of change, comorbidities, anthropometric and body composition measures, smoking habit, obesity, drug treatments, Pfeffer's FAQ, World Health Organization QoL, Mediterranean diet adherence, physical activity. | The combined used of smartphone app and smartband for 3 months did not result in lifestyle changes related to the mount of physical activity or eating habits and other clinical parameters compared to brief lifestyle advice. Only differences were reported at the cognitive level, where a slight improvement was observed in the CDT score. |
| (Sanabria et al., 2018 [77]) | Memory Clinic of Fundació ACE, *Institut Català de Neurociències Aplicades* (Barcelona, Spain) | Determine whether performance on S-FNAME was associated with Aβ burden in subjective cognitive decline (SCD) individuals. | Cross-sectional | 200 | >49<br>M: 65.81 (7.28) | F: 62% (125)<br>M: 38% (75) | SCD | S-FNAME, WAIS-III vocabulary test, MMSE, MFE | Age, sex, years of education. | S-FNAME performance, a face-name associative memory test, is related to higher Aβ deposition in healthy adults with SCD and that the SFN-N may be the most sensitive subtest at detecting Aβ burden |
| (Sánchez-Benavides et al., 2016 [78]) | NEURONORMA sample, Hospital del Mar Research Institute, Barcelona, Spain. | Provide several reliable change indices (RCI) for a 1-year follow-up in Spanish Neuropsychological tests. | Cohort | 122 | >50<br>M: 64.5 (8.7) | F: 84.69% (103)<br>M: 15.31% (19) | Cognitively normal | MMSE, MIS, DS, Corsi's Test, TMT, Symbol Digit Modalities Test, BNT, Token test, Subtests of VOSP battery, JLOT, ROCF, FCSRT, VFT, Stroop test and | Sex, age, education years. | Significant improvements were observed in variables related to memory, both verbal and visual, visuospatial function, and the completion time of complex problems. |

| | | | | | | | | | | |
|---|---|---|---|---|---|---|---|---|---|---|
| | | | | | | | | Tower of London Drexel version | | |
| (Sánchez-Benavides et al., 2014 [79]) | 9 hospitals in different regions of Spain. | Characterise the neuropsychological and neuroimaging profiles of MCI and AD patients and compare them with healthy subjects. | Cross-sectional | 535 | 50-85<br>M: 64.9 (9.3) | M: 39.8% (213)<br>F: 60.2% (322) | 356 cognitively healthy, 79 MCI and 100 AD. | MMSE and NEURONORMA neuropsychological battery: DS, Corsi's Tests, TMT, Symbol Digit Modalities Test, BNT, Token Test, ROCF, FCSRT, SVF, PVF, Stroop interference test, Tower of London Drexel version and Visual Object and Spatial Perception Battery. | Age, education years, Interview for Deterioration of Daily living activities in Dementia (IDD), Modified Hachinski Ischaemia Score, Hamilton Depression Rating Scale, blood tests and imaging (CT or MRI), APOE-E4 alleles. | Neuropsychological and imaging profiles were described. Globally, neuropsychological measures observed larger differences than MRI measures when patients and healthy subjects were compared. |
| (Santabárbara et al., 2019 [80]) | Zaragoza Dementia Depresion Project (ZARADEMP) | Examine the association between the principal lifestyle occupation and the incidence of AD. | Cohort | 3883 | >55<br>M: 70.86 (8.9) | F: 55.4% (2161)<br>M: 44.6% (1722) | Community based cohort | MMSE | Age, sex, educational level, cohabitation, comorbidities, BMI. | Farmers had a lower risk of AD, reinforcing the importance of lifetime occupation in the risk of AD at older ages. |
| (Soldevila-Domenech et al., 2021 [81]) | All participants were recruited in the context of the PENSA multimodal intervention clinical trial. Hospital del mar, Barcelona. | Explore the impact of the Spanish COVID-19 strict home confinement on mental health and cognition in non-infected subjects. | Prospective | 16 | 60-80<br>M: 65.8 (3.75) | M: 37.5% (6)<br>F: 62.5% (10) | SCD and *APOE* 3/4 carriers. | MoCA | Age, gender, marital status, occupation, GHQ-28, anxiety, depression, anthropometry, physical activity, sleep, mood trajectories. | Those with lower mood during confinement experienced a decline in their mood after confinement. Cognition did not change. |
| (Valiente-Barroso et al., 2015 [82]) | Tres Mares Hospital, Reinosa, Cantabria, Spain. | Analyse the potential impact of factors associated with diabetes on certain mental processes related to cognitive impairment. | Cross-sectional | 59 | 50-85<br>M: 70.98 (10.24) | M: 44.1% (26)<br>F: 55.9% (33) | Diabetes, cognitively healthy | MMSE, Stroop interference test, TMT-a, WAIS DS forward and backward | Age, education years, BMI, diabetes type and years, depression, cardiopathy, hypercholesterolaemia, HBP, exercise. | Cognitive performance showed an inverse relationship to age and cardiopathy, while years of education and regular physical activity appeared as neuroprotective factors. Diabetes may be regarded as a risk factor for the development of CI, but further research is needed. |

ADL: Activities of daily living**,** APOE-E4: Apolipoprotein-E4, BMI: Body Mass Index, BNT: Boston Naming Test, CAMCOG-R: Cambridge Cognitive Assessment Revised, CD: cognitive decline, CDT: Clock Drawing Test, CI: cognitive impairment, CVA: Cardiovascular accident, CVLT: California Verbal Learning Test, DS: Digit span, FAQ: Functional Activities Questionnaire, FCSRT: Free and Cued Selective Reminding Test, FNAME: Face Name Associative Memory

Exam, GDS: Geriatric Depression Scale, GHQ-28: General Health Questionnaire, HBP: High blood pressure, JLOT: Judgement of Line Orientation Test, MCI: Mild cognitive impairment, MFE: Memory Failures of everyday, MIS: Memory Impairment Screen, MMSE: Mini-mental state examination, MNA: Mini Nutritional Assessment, MoCA: Montreal Cognitive Assessment, NBACE: Neuropsychological Battery from Fundació ACE, PVF: Phonetic verbal fluency, QoL: Quality of Life, RCT: Randomized controlled trial, ROCF: Rey-Osterrieth complex figure, SCI: Subjective cognitive impairment, SMC: subjective memory complaints, SPMSQ: Short Portable Mental State Questionnaire, STICS-m: Spanish Telephone Interview for Cognitive Status-modified, SVF: semantic verbal fluency, TMT: Trail Making Test, VFT: Verbal fluency tests (SVF and PVF), VOSP: Visual Object and Spatial Perception, WAIS-III: Weschler Adult Intelligence Scale- third Edition, WMS-III: Weschler Memory Scale- Third Edition.

## REFERENCES


51. Alegret, M.; Peretó, M.; Pérez, A.; Valero, S.; Espinosa, A.; Ortega, G.; Hernández, I.; Mauleón, A.; Rosende-Roca, M.; Vargas, L.; et al. The Role of Verb Fluency in the Detection of Early Cognitive Impairment in Alzheimer's Disease. *J. Alzheimer's Dis.* **2018**, *62*, 611–619, https://doi.org/10.3233/jad-170826.
52. Alegret, M.; Rodríguez, O.; Espinosa, A.; Ortega, G.; Sanabria, A.; Valero, S.; Hernández, I.; Rosende-Roca, M.; Vargas, L.; Abdelnour, C.; et al. Concordance between Subjective and Objective Memory Impairment in Volunteer Subjects. *J. Alzheimer's Dis.* **2015**, *48*, 1109–1117, https://doi.org/10.3233/jad-150594.
53. Bayes-Marin, I.; Fernández, D.; Lara, E.; Martín-María, N.; Miret, M.; Moreno-Agostino, D.; Ayuso-Mateos, J.L.; Sanchez-Niubo, A.; Haro, J.M.; Olaya, B. Trajectories of Immediate and Delayed Verbal Memory in the Spanish General Population of Middle-aged and Older Adults. *Brain Sci.* **2020**, *10*, 249, https://doi.org/10.3390/brainsci10040249.
54. Climent, M.T.; Ballesteros, C.; Colomer, V.; Botella, P.; Moreno, L. Deterioro cognitivo y horas de sueño en mayores de 65 años no institucionalizados: estudio en farmacia comunitaria. *Farm. Comunitarios* **2015**, *7*, 25–30, https://doi.org/10.5672/fc.2173-9218.(2015/vol7).002.04.
55. Delgado-Lima, A.H.; Bouhaben, J.; Martínez-Zujeros, S.; Pallardo-Rodil, B.; Gómez-Pavón, J.; Delgado-Losada, M.L. Could olfactory identification be a prognostic factor in detecting cognitive impairment risk in the elderly? *GeroScience* **2023**, *45*, 2011–2025, https://doi.org/10.1007/s11357-023-00779-5.
56. Fernández-Matarrubia, M.; Goni, L.; Rognoni, T.; Razquin, C.; Fernández-Lázaro, C.I.; Bes-Rastrollo, M.; Martínez-González, M.Á.; Toledo, E. An Active Lifestyle Is Associated with Better Cognitive Function Over Time in *APOE* ε4 Non-Carriers. *J. Alzheimer's Dis.* **2021**, *79*, 1257–1268, https://doi.org/10.3233/jad-201090.
57. Ferreira, D.; Bartrés-Faz, D.; Nygren, L.; Rundkvist, L.J.; Molina, Y.; Machado, A.; Junqué, C.; Barroso, J.; Westman, E. Different reserve proxies confer overlapping and unique endurance to cortical thinning in healthy middle-aged adults. *Behav. Brain Res.* **2016**, *311*, 375–383, https://doi.org/10.1016/j.bbr.2016.05.061.
58. Galbete, C.; Toledo, E.; Toledo, J.B.; Bes-Rastrollo, M.; Buil-Cosiales, P.; Marti, A.; Guillén-Grima, F.; Martínez-González, M.A. Mediterranean diet and cognitive function: The sun project. *J. Nutr. Heal. Aging* **2015**, *19*, 305–312, https://doi.org/10.1007/s12603-015-0441-z.
59. Villanueva, M.Z.G.; Valiente, J.M.U.; Zarranz, A.R.-A. Quality of Life in an Adult Population of More than 60 Years of Age without Cognitive Impairment. *Dement. Geriatr. Cogn. Disord. Extra* **2014**, *4*, 355–363, https://doi.org/10.1159/000365505.
60. García-Garro, P.A.; Hita-Contreras, F.; Martínez-Amat, A.; Achalandabaso-Ochoa, A.; Jiménez-García, J.D.; Cruz-Díaz, D.; Aibar-Almazán, A. Effectiveness of A Pilates Training Program on Cognitive and Functional Abilities in Postmenopausal Women. *Int. J. Environ. Res. Public Heal.* **2020**, *17*, 3580, https://doi.org/10.3390/ijerph17103580.
61. Gil-Peinado, M.; Alacreu, M.; Ramos, H.; Sendra-Lillo, J.; García, C.; García-Lluch, G.; de Coca, T.L.; Sala, M.; Moreno, L. The A-to-Z factors associated with cognitive impairment. Results of the DeCo study. *Front. Psychol.* **2023**, *14*, 1152527, https://doi.org/10.3389/fpsyg.2023.1152527.
62. Goni, L.; Fernández-Matarrubia, M.; Romanos-Nanclares, A.; Razquin, C.; Ruiz-Canela, M.; Martínez-González, M.Á.; Toledo, E. Polyphenol intake and cognitive decline in the Seguimiento Universidad de Navarra (SUN) Project. *Br. J. Nutr.* **2020**, *126*, 43–52, https://doi.org/10.1017/s000711452000392x.
63. González, M.F.; Facal, D.; Juncos-Rabadán, O.; Yanguas, J. Socioeconomic, emotional, and physical execution variables as predictors of cognitive performance in a Spanish sample of middle-aged and older community-dwelling participants. *Int. Psychogeriatrics* **2017**, *29*, 1669–1680, https://doi.org/10.1017/s1041610217001144.
64. Lara, E.; Olaya, B.; Garin, N.; Ayuso-Mateos, J.L.; Miret, M.; Moneta, V.; Haro, J.M. Is cognitive impairment associated with suicidality? A population-based study. *Eur. Neuropsychopharmacol.* **2015**, *25*, 203–213, https://doi.org/10.1016/j.euroneuro.2014.08.010.
65. Lojo-Seoane, C.; Facal, D.; Guàrdia-Olmos, J.; Pereiro, A.X.; Campos-Magdaleno, M.; Mallo, S.C.; Juncos-Rabadán, O. Cognitive reserve and working memory in cognitive performance of adults with subjective cognitive complaints: longitudinal structural equation modeling. *Int. Psychogeriatrics* **2019**, *32*, 515–524, https://doi.org/10.1017/s1041610219001248.
66. López, M.; Ríos, A.; Romaguera, D.; Martínez-González, M.Á.; Fernández-Aranda, F.; Salas-Salvadó, J.; Corella, D.; Fitó, M.; Vioque, J.; Alonso-Gómez, Á.M.; et al. Asociación entre índice tobillo-brazo y rendimiento cognitivo en participantes del estudio PREDIMED-Plus: estudio transversal. *REC: Interv. Cardiol. (English Ed.* **2021**, *74*, 846–853, https://doi.org/10.1016/j.recesp.2020.06.031.
67. López-Higes, R.; Prados, J.M.; Rubio-Valdehita, S.; Rodríguez-Rojo, I.; de Frutos-Lucas, J.; Montenegro, M.; Montejo, P.; Prada, D.; Losada, M.L.D. Factors Explaining Language Performance After Training in Elders With and Without Subjective Cognitive Decline. *Front. Aging Neurosci.* **2018**, *10*, 264, https://doi.org/10.3389/fnagi.2018.00264.

68. Muñoz-Garach, A.; Cornejo-Pareja, I.; Martínez-González, M.Á.; Bulló, M.; Corella, D.; Castañer, O.; Romaguera, D.; Vioque, J.; Alonso-Gómez, Á.M.; Wärnberg, J.; et al. Milk and Dairy Products Intake Is Related to Cognitive Impairment at Baseline in Predimed Plus Trial. *Mol. Nutr. Food Res.* **2021**, *65*, e2000728, https://doi.org/10.1002/mnfr.202000728.
69. Muñoz-García, M.; Cervantes, S.; Razquin, C.; Guillén-Grima, F.; Toledo, J.B.; Martínez-González, M.Á.; Toledo, E. Validation study of a Spanish version of the modified Telephone Interview for Cognitive Status (STICS-m). *Gac. Sanit.* **2019**, *33*, 415–420, https://doi.org/10.1016/j.gaceta.2018.05.004.
70. Muñoz-García, M.I.; Martínez-González, M.A.; Martín-Moreno, J.M.; Razquin, C.; Cervantes, S.; Guillén-Grima, F.; Toledo, E. Sugar-sweetened and artificially-sweetened beverages and changes in cognitive function in the SUN project. *Nutr. Neurosci.* **2019**, *23*, 946–954, https://doi.org/10.1080/1028415x.2019.1580919.
71. Muñoz-García, M.I.; Martínez-González, M.A.; Razquin, C.; Fernández-Matarrubia, M.; Guillén-Grima, F.; Toledo, E. Exploratory dietary patterns and cognitive function in the "Seguimiento Universidad de Navarra" (SUN) Prospective Cohort. *Eur. J. Clin. Nutr.* **2021**, *76*, 48–55, https://doi.org/10.1038/s41430-021-00922-5.
72. Ni, J.; Nishi, S.K.; Babio, N.; Martínez-González, M.A.; Corella, D.; Castañer, O.; Martínez, J.A.; Alonso-Gómez, Á.M.; Gómez-Gracia, E.; Vioque, J.; et al. Dairy Product Consumption and Changes in Cognitive Performance: Two-Year Analysis of the PREDIMED-Plus Cohort. *Mol. Nutr. Food Res.* **2022**, *66*, e2101058, https://doi.org/10.1002/mnfr.202101058.
73. Nishi, S.K.; Babio, N.; Gómez-Martínez, C.; Martínez-González, M.Á.; Ros, E.; Corella, D.; Castañer, O.; Martínez, J.A.; Alonso-Gómez, Á.M.; Wärnberg, J.; et al. Mediterranean, DASH, and MIND Dietary Patterns and Cognitive Function: The 2-Year Longitudinal Changes in an Older Spanish Cohort. *Front. Aging Neurosci.* **2021**, *13*, 782067, https://doi.org/10.3389/fnagi.2021.782067.
74. Nishi, S.K.; Babio, N.; Paz-Graniel, I.; Serra-Majem, L.; Vioque, J.; Fitó, M.; Corella, D.; Pintó, X.; Bueno-Cavanillas, A.; Tur, J.A.; et al. Water intake, hydration status and 2-year changes in cognitive performance: a prospective cohort study. *BMC Med.* **2023**, *21*, 1–17, https://doi.org/10.1186/s12916-023-02771-4.
75. Ramos, H.; Moreno, L.; Pérez-Tur, J.; Cháfer-Pericás, C.; García-Lluch, G.; Pardo, J. CRIDECO Anticholinergic Load Scale: An Updated Anticholinergic Burden Scale. Comparison with the ACB Scale in Spanish Individuals with Subjective Memory Complaints. *J. Pers. Med.* **2022**, *12*, 207, https://doi.org/10.3390/jpm12020207.
76. Razquin, C.; Menéndez-Acebal, C.; Cervantes, S.; Martínez-González, M.A.; Vázquez-Ruiz, Z.; Martínez-González, J.; Guillén-Grima, F.; Toledo, E. Hypertension and changes in cognitive function in a Mediterranean population. *Nutr. Neurosci.* **2020**, *25*, 612–620, https://doi.org/10.1080/1028415x.2020.1788773.
77. Sanabria, A.; Alegret, M.; Rodriguez-Gomez, O.; Valero, S.; Sotolongo-Grau, O.; Monté-Rubio, G.; Abdelnour, C.; Espinosa, A.; Ortega, G.; Perez-Cordon, A.; et al. The Spanish version of Face-Name Associative Memory Exam (S-FNAME) performance is related to amyloid burden in Subjective Cognitive Decline. *Sci. Rep.* **2018**, *8*, 1–9, https://doi.org/10.1038/s41598-018-21644-y.
78. Sánchez-Benavides, G.; Peña-Casanova, J.; Casals-Coll, M.; Gramunt, N.; Manero, R.M.; Puig-Pijoan, A.; Aguilar, M.; Robles, A.; Antúnez, C.; Frank-García, A.; et al. One-Year Reference Norms of Cognitive Change in Spanish Old Adults: Data from the NEURONORMA Sample. *Arch. Clin. Neuropsychol.* **2016**, *31*, 378–388, https://doi.org/10.1093/arclin/acw018.
79. Sánchez-Benavides, G.; for the NEURONORMA Study Team; Peña-Casanova, J.; Casals-Coll, M.; Gramunt, N.; Molinuevo, J.L.; Gómez-Ansón, B.; Aguilar, M.; Robles, A.; Antúnez, C.; et al. Cognitive and Neuroimaging Profiles in Mild Cognitive Impairment and Alzheimer's Disease: Data from the Spanish Multicenter Normative Studies (NEURONORMA Project). *J. Alzheimer's Dis.* **2014**, *41*, 887–901, https://doi.org/10.3233/jad-132186.
80. Santabárbara, J.; Gracía-Rebled, A.C.; López-Antón, R.; Tomás, C.; Lobo, E.; Marcos, G.; Lobo, A. The effect of occupation type on risk of Alzheimer's disease in men and women. *Maturitas* **2019**, *126*, 61–68, https://doi.org/10.1016/j.maturitas.2019.05.008.
81. Soldevila-Domenech, N.; Forcano, L.; Boronat, A.; Lorenzo, T.; Piera, I.; Puig-Pijoan, A.; Mateus, J.; Gómez, J.M.G.d.E.; Knezevic, I.; Soteras, A.; et al. Effects of COVID-19 Home Confinement on Mental Health in Individuals with Increased Risk of Alzheimer's Disease. *J. Alzheimer's Dis.* **2021**, *79*, 1015–1021, https://doi.org/10.3233/jad-201408.
82. Valiente-Barroso, C.; Alvarado-Izquierdo, J.M.; García, E.G. Clinical and Sociodemographic Factors Associated with Cognitive Impairment and Neuroprotection in Diabetes Patients. *Span. J. Psychol.* **2015**, *18*, E65, https://doi.org/10.1017/sjp.2015.61.
